# Identification and Immune Landscape Analysis of Fatty Acid Metabolism-related Genes Associated with Rheumatoid Arthritis

**DOI:** 10.7150/ijms.121972

**Published:** 2026-01-01

**Authors:** Zhongyang Zhou, Rong Luo, Lan Shao

**Affiliations:** The Center for Translational Medicine, The First Affiliated Hospital, Sun Yat-Sen University, Guangzhou, P.R. China 510080.

**Keywords:** bioinformatic analysis, rheumatoid arthritis, synovial, fatty acid metabolism, PDK1, XBP1, ACACB

## Abstract

**Background:** Dysfunction of fatty acid metabolism plays a critical role in the pathogenesis of Rheumatoid Arthritis (RA). This study aimed to screen for Hub genes involved in fatty acid metabolism that contribute to the inflammatory state of RA synovium.

**Methods:** Four mRNA microarray datasets for RA were integrated into an expression matrix as a test dataset. One RNA-seq and five microarray datasets were preprocessed as validation datasets. Immune cell infiltration combined with Weighted Gene Co-expression Network Analysis (WGCNA) were used to feature infiltrated cells and their correlation with candidate genes in RA. Five machine learning algorithms were applied to Hub genes screening. Temporal, immuno-efficacy, drug target prediction, molecular docking, ceRNA, and transcription factors networks analyses were conducted to elucidate the association of the Hub genes with RA. Immunofluorescence assay was performed in Collagen-Induced Arthritis (CIA) mouse, and qPCR and Western blot were applied to TNFα or IL-6 treated MH7A cells to reveal the potential roles of the proinflammatory cytokines on Hub genes expression in RA synovium.

**Results:** Three Hub genes with better diagnostic efficiency were screened, with *PDK1* and *XBP1* up-regulated and *ACACB* down-regulated in RA. These genes were associated with immune cells infiltration and immuno-efficacy in RA, and their expression patterns showed time-dependent characteristics during disease progression. Mechanistically, MALAT1, NEAT1 and FOXC1 were involved in the regulation of PDK1, XBP1 and ACACB expression, and TNFα or IL-6 treatment mimicked their expression phenotypes in RA.

**Conclusion:** Our study identified *PDK1*, *XBP1* and *ACACB* as the Hub genes from the fatty acid metabolic pathway and indicated that PDK1, XBP1 and ACACB might play key roles in the pathogenesis of RA synovium.

## Introduction

Rheumatoid Arthritis (RA) is a chronic autoimmune disease, and its basic pathological change in the early stage is synovial inflammation 1, 2. The destruction of synovial membrane of joints occurs within 3 months of the onset of RA, and it is difficult to reverse once bone erosion and joint deformity occur 2, 3. Therefore, early diagnosis and treatment at an early stage of RA are crucial. Joint inflammation in RA leads to changes in the energy metabolism of joint cells, especially synovial cells and chondrocytes, which require more energy to support cell proliferation, differentiation, and synthesis of extracellular matrix to respond to inflammation and repair bone damage 4-9. Multiple metabolic pathways, such as glycolysis and fatty acid metabolism, are dysregulated in the RA synovium 4-7, 10.

Fatty acid metabolism (FAM) contributes to the synovial inflammatory environment formation, affecting both resident immune cells 4, 5, 11, 12 and fibroblast-like synovial cells (FLS) 7, 13, 14. T cells from patients with RA tend to increase the uptake and oxidation of fatty acids, which is related to the overactivation of T cells and the intensification of the inflammatory response 11, 15. Specific fatty acids, such as unsaturated fatty acids, can affect the proliferation, apoptosis and immune-modulatory function of T cells 16-18. The up-regulated expression of fatty acid synthase (FASN) and long-chain fatty acid-CoA synthase 4 (ACSL4) in Regulatory T cells (Treg) are associated with the inhibitory function of Treg 19, 20. Moreover, changes in fatty acid metabolism in RA are related to the recruitment of T helper cell 17 (Th17) and the generation of osteoclasts, thus aggravating joint inflammation and bone erosion 21. In addition, the synovial microenvironment of RA has been shown to induce the maturation and metabolic reprogramming of immune cells, such as macrophage and CD11c^+^ dendritic cells, which exhibit increased expression of genes associated with fatty acid metabolism 22.

Increasing evidence shows that fatty acid metabolic pathways of RA-FLS are involved in the inflammatory environment formation of RA synovium 7, 13, 14. Leptin-driven fatty acid β-oxidation (FAO) activation enhances the proinflammatory characteristics of FLS in RA, and the proinflammatory phenotype of RA-FLS can be inhibited by silencing leptin expression or with etomoxir, a well-known FAO pathway inhibitor 23. On the other hand, synovial tissue in inflamed joints is a rich source of proinflammatory cytokines 24, 25. Fatty acid metabolism also needs to adopt to and be modified by this inflammatory microenvironment. Specially, TNFα and *Interleukin* 6 (IL-6) are dominant proinflammatory cytokines within the inflamed bone microenvironment in RA 26-28. However, the roles of these cytokines in the regulation of fatty acid metabolism in RA remain unclear.

In the current study, we conducted bioinformatics analyses and utilized machine learning algorithms to explore Hub genes associated with the fatty acid metabolic pathway in the synovial membrane of RA. The expression patterns of the Hub genes in RA synovium were elucidated through protein-protein interaction (PPI), temporal and immune cell infiltration analyses. Moreover, immuno-efficacy evaluation, drug target prediction and transcription factor network prediction were performed to provide hints for possible therapeutic application and related regulatory mechanism. Further biological investigation using Collagen-Induced Arthritis mouse (CIA) and MH7A cells implicated the potential regulation mechanism of the Hub genes expression in RA synovium. These insights from our study would be valuable for further understanding the impact of the fatty acid metabolic pathway on RA pathogenesis.

## Material and Methods

### Data acquisition and preprocessing

The overall workflow of this study is shown in Supplementary Figure S1. Four mRNA microarray datasets of RA synovium (GSE12021, GSE55235, GSE55457, GSE77298) generated on the Affymetrix HG-U133A (GPL96) and HG-U133 Plus 2.0 (GPL570) platforms were downloaded from GEO database 29-33. The GPL570 platform contained probes that were not present in GPL96, therefore, only the probes common to both platforms were retained to ensure comparability. Background correction and normalization were performed using the Robust Multi-array Average (RMA) algorithm to generate gene-level expression values. Probe IDs were converted to official gene symbols using the platform-specific annotation packages (hgu133a.db for GPL96 and hgu133plus2.db for GPL570). To handle the common issue of probe-to-gene symbol, the following strategy was employed: for probe groups corresponding to multiple genes, the first one was selected for retention; for genes having multiple probes, the average expression value of the probes was taken. By matching probe IDs and gene Symbols, probe expression matrices were converted into gene expression matrices. Gene expression values were log2-transformed. The normalizeBetweenArrays function of the limma package was applied for intra-group data normalization processing. The intersect function was used to extract common genes. The cbind function was used to merge the gene expression matrices of the four datasets. The integrated dataset was then processed for batch effect correction using the ComBat algorithm from the sva R package, with the dataset source as the batch variable. Parametric empirical Bayes framework of ComBat was used to adjust for location and scale shifts in the data across batches. The biological condition (RA vs. HC) was included as a model covariate to preserve the biological signal of interest. Principal component analyses (PCA) were performed both before and after batch effect correction and visualized by PCA plots for confirmation. The integrated expression matrix obtained after background correction and batch effect correction was considered as the test dataset. The limma package was used to analyze differentially expressed genes (DEGs) using the threshold of adj.p-value < 0.05 and |logFC| > 1 34. The ggplot2 package and pheatmap package were employed to visualize the results with volcano plot and heatmap. The RNA-seq dataset (GSE89408) was downloaded and subjected to the Counts and FPKM numerical transformation using Counts2FPKM package for normalization to obtain accurate gene expression data. Differential expression analysis and statistical test were performed using DESeq2, resulting in the generation of gene matrix as the validation dataset 35. Five other mRNA microarray datasets with clinical samples from different immunotherapy drug treatments were downloaded for background correction, and their expression matrices were extracted as additional validation datasets. Differential expression analyses were performed using the DESeq2 package for the validation datasets mentioned above. GEO information included in this study is provided in Supplementary Table S1.

This study downloaded the "fatty acid metabolism" gene sets from the MSigDB database (https://www.gsea-msigdb.org/gsea/msigdb/human/search.jsp). A total of 4 most relevant gene sets were selected: HALLMARK_FATTY_ACID_METABOLISM, KEGG_FATTY_ACID_METABOLISM, REACTOME_FATTY_ACID_METABOLISM, and GOBP_FATTY_ACID_METABOLIC_PROCESS. A total of 536 genes related to fatty acid metabolism (FAM) were identified, and 24 FAM_DEGs (FAM-related DEGs) were obtained by intersection with DEGs from the test dataset.

All research involving human databases was approved by the Medical Ethics Committee of first affiliated hospital of *Sun Yat-Sen* University.

### Enrichment and protein-protein interaction network analysis

Gene Ontology (GO) and Kyoto Encyclopedia of Genes Genomes (KEGG) analyses were conducted using the Clusterprofiler package. The thresholds were set as p value Cut off < 0.05 and q value Cut off < 0.05. The dotplot function was utilized to create bubble plots, and the circlize package was used to generate circle plots for visualizing the results of the enrichment analyses. Gene Set Enrichment Analysis (GSEA) was performed based on the entire gene set in MigDB, and the gseaplot function was used to visualize the up- and down-regulated pathways. The DEGs were imported into the STRING database (http://string-db.org) to construct the PPI network followed by the visualization using Cytoscape software. The CytoHubba plugin and the Maximum Clique Centrality (MCC) topological algorithm were used to calculate Node Scores as criteria, and the top ten DEGs were selected as Hub candidate genes.

### Immune infiltration analysis using CIBERSORT

The requirements of the CIBERSORT algorithm for input matrix are as follows: (1) no missing or negative values; (2) log conversion is not performed; (3) affymetrix chips use RMA standardization, Illumina's Beadchip and Agilen monochrome chips use limma processing; (4) for RNA-seq sequencing data, either FPKM or TPM numerical types are applicable 36, 37. The original data of the test dataset was downloaded, and the RMA function was used for background correction, data standardization, expression value calculation, and log2 transformation and ID transformation. The ComBat function of the sva package was applied for batch effect correction. Permutation tests were used to evaluate the reliability of the results. The number of permutations parameter was set as perm = 1000 to enhance the credibility of cell proportion estimation. DEGs in each dataset were screened according to the threshold of adj.p-value < 0.05 and |logFC| > 1, and the differential gene expression matrix of the test dataset was obtained for immune cell infiltration analysis.

The relative proportions of immune cell types in each sample were quantified using the CIBERSORT algorithm with the LM22 signature matrix. The algorithm reported a p-value for each sample, which reflected that the result was not due to random chance. A p-value < 0.05 was generally considered indicative of a reliable estimate for that individual sample and was not directly equivalent to the statistical significance of differences in cell proportions between groups. For group comparisons (RA vs. HC), the differences in immune cell fractions were assessed using the Mann-Whitney U test, and a p-value < 0.05 was considered statistically significant. The observed non-significant p-values for some inter-groups might reflect the substantial biological heterogeneity inherent in RA synovial tissue. The immune cells with insignificant p-values were excluded. The Wilcoxon test was employed to identify immune cells with significant differences between RA and healthy controls (HCs), which were selected as the phenotypic data for subsequent Weighted Gene Co-expression Network Analysis (WGCNA) 38.

### Weighted gene co-expression network analysis

The WGCNA package was used to identify genes associated with immune cell infiltration. The outliers of gene expression matrix were identified using hierarchical clustering. Spearman correlation coefficients between genes were calculated and the similarity matrix was constructed. A suitable soft threshold was selected to transform the similar matrix into an adjacency matrix, which was further transformed into a topological overlap matrix (TOM). Dynamic pruning tree recognition module was used, and clustering was based on 1-TOM distance. The predominant immune infiltrating cells in the RA microenvironment were identified. The module genes with the highest correlation with RA, fatty acid-related genes and differentially expressed genes were extracted and intersected as Hub candidate genes for subsequent correlation analysis. Spearman rank correlation coefficient analysis was performed on the significantly different immune infiltrates and the thirteen Hub candidate genes using the corrplot package, with the cor.mtest function conducting a significance test on the correlation matrix based on Hotelling-Pabst test.

### Machine Learning algorithms screening and validation for Hub genes

Five machine learning algorithms were combined to screen Hub genes: LASSO (Least Absolute shrinkage and selection operator) 39, SVM-RFE (Support vector machine recursive feature elimination) 40, RF (Random Forest), XGBoost (limit gradient lifting eXtreme Gradient Boosting) 41 and Boruta 42, 43. Each machine learning algorithm has its own inherent biases and assumptions. By combining multiple algorithms, the biases can be reduced, and the result of feature selection can be more reliable. The Hub genes were internally and externally validated using both the test dataset and validation dataset. The ggboxplot function was used to visualize the gene expression levels of samples from patients with RA and HCs, and the Wilcoxon test was used to calculate the significance.

### Diagnostic efficiency evaluation and temporal analysis

The Receiver Operating Characteristic (ROC) curves and the Area Under the Curve (AUC) values were used to evaluate the diagnostic efficiency of the Hub genes between RA and HC. The pROC package was utilized to generate ROC curves and calculate AUC values, and the roc.test function was used for statistical analyses.

Temporal analysis is a method for analyzing the temporal trends of transcription changes using Mfuzz package in R, which employs the core algorithm based on Fuzzy C-Means Clustering (FCM) for soft clustering. Mfuzz assigns each gene a membership value to each cluster, reflecting the biological reality that genes can participate in multiple coordinated processes. Temporal analysis was conducted using six disease progression time points from RA to HC in the validation dataset GSE89408, including normal, osteoarthritis, arthralgia, undifferentiated arthritis, RA (early) and RA (established) stages. The data was filtered to remove genes with standard deviation of 0 to ensure each time course was comparable. The critical fuzziness parameter “m” was set to 1.711549, which was determined using the mestimate function, ensuring a balance between cluster overlap and distinctness. The optimal number of clusters (five) was determined using the vegan package, which provided a clear separation of major temporal trends while minimizing overly complex patterns. The Mfuzz function was applied to process the time node information of the samples and the expression profile information of DEGs, examining the number of DEGs in each cluster and the cluster to which each DEG belonged. Genes were assigned to a core cluster based on their highest membership value. A cluster was considered to represent a robust time-dependent pattern if its centroid exhibited a clear directional or phased trajectory and contained genes with high membership values (typically > 0.5). The Mfuzz.plot was used to visualize clustering results, and the cluster hierarchy to which the Hub genes belonged was extracted to analyze their temporal expression patterns.

### Immuno-efficacy evaluation and molecular docking analysis

Five mRNA microarray datasets containing clinical samples with different immunotherapy treatments were used as validation datasets. The GSE15258 dataset was divided into TNF antibody response and non-response groups. The GSE37107 dataset was divided into Rituximab response and non-response groups. The GSE58795 dataset was divided into groups treated with Infiximab and placebo. The GSE68215 dataset was divided into groups with low and non-low disease activity. The GSE45867 dataset was grouped into before- and after-controls for Methotrexate and Abatacept, respectively. The ggboxplot function was used to plot the expression boxplots for the Hub genes across different immunotherapy treatments, and the Wilcoxon test was used to assess the significance.

The DSigDB (https://dsigdb.tanlab.org/DSigDBv1.0/) and Enrichr (https://maayanlab. cloud /Enrichr/#) online tools were used to predict potential drug molecules targeting the Hub genes. Small drug molecules with strong binding ability to the three Hub genes (*PDK1*, *XBP1* and *ACACB*) were selected based on the binding p-values and scores for molecular docking analysis. The protein PDB numbers and corresponding tertiary structures for the Hub genes were downloaded from the PDB database (https://www.rcsb.org/). The small molecule CID numbers and structures were obtained from the PubChem database (https://pubchem.ncbi.nlm.nih.gov/). Autodock Tools and PyMOL software were used for protein-ligand docking analysis and visualization.

### ceRNA and transcription factor network analysis

The ceRNA network is an emerging gene regulatory mechanism that regulates variably spliced RNAs through competitive binding of endogenous microRNAs (miRNAs) to influence each other's expression. The ceRNA network of the Hub genes was analyzed using the miRDB (https://mirdb.org/index.html) and ENCORI (https://rnasysu.com/encori/) websites. The ggalluvial package was employed to draw a mulberry map to visualize the lncRNA-miRNA-mRNA regulatory network of the Hub genes.

NetworkAnalyst (https://www.networkanalyst.ca/) was used for transcription factor prediction and interaction network construction. The JASPAR database was employed to predict the transcription factors associated with the Hub genes.

### Cell culture and treatment

The human synovial fibroblast-like cell line MH7A was obtained from the American Type Culture Collection. The MH7A cells were cultured in RPMI 1640 (Invitrogen, Carlsbad, CA, USA) supplemented with 10% fetal bovine serum (Invitrogen) and 1% penicillin/streptomycin (Invitrogen) at 37 °C in a humidified atmosphere with 5% CO_2_. The cells were treated with 50 ng/mL TNFα or 10 ng/mL IL-6 (Peprotech, Rocky Hill, NJ, USA) for 4 or 8 hours before being harvested for experimental assays.

### RNA isolation and quantitative real-time PCR (qPCR)

The total RNA was extracted from 1 × 10^5^ cells followed by cDNA synthesis using the BeyoRT^TM^II First Strand cDNA Synthesis Kit (Beyotime, Shanghai, China). RT-qPCR using Universal SYBR qPCR Master Mix was performed on the ABI StepOne PLUS Real-Time PCR System (Applied Biosystems, USA). The primer sequences, for *PDK1* were 5'-CTGTGATACGGATCAGAAACCG-3' and 5'-TCCACCAAACAATAAAGAGTGCT-3'; for *XBP1* were 5'-CCCTCCAGAACATCTCCCCAT-3' and 5'-ACATGACTGGGTCCAAGTTGT-3'; for *ACACB* were 5'-AGAAGACAAGAAGCAGGCAAAC-3' and 5'-GTAGACTCACGAGATGAGCCA-3', for *GAPDH* were 5'-AAGGTCATCCCAGAGCTG AA-3' and 5'-CTGCTTCACCACCTTCTTGA-3'. Relative mRNA expression level was calculated using the 2^-ΔΔCt^ method, normalizing to the housekeeping gene *GAPDH*.

### Western blot

The proteins were extracted using RIPA buffer (Beyotime, Shanghai, China) supplemented with PMSF (Beyotime). After quantification, the extracted samples were separated by SDS-PAGE and transferred to polyvinylidene fluoride membranes. The membranes were blocked with 5% Bovine Serum Albumin for 1h and then incubated with primary antibodies at 4 °C for 12 h. The membranes were probed with anti-ACACB (1:1000, Cat# DF7980, Affinity, CA, USA) and anti-PDK1 (1:1000, Cat# DF4365, Affinity), and anti-XBP1 (1:1000, Cat# sc-8015, Santa Cruz, CA, USA) and anti-Actin (1:1000, Cat# sc-58673, Santa Cruz) primary antibodies. The membranes were subsequently incubated with secondary antibodies for 1 h at room temperature and developed with a chemiluminescent detection system (Applied Biosystems, USA). The protein expression levels were quantified using Image J 9.0 software (NIH, USA) by measuring the band intensities and normalizing them to those of Actin.

### Immunofluorescence staining

The synovium tissues from control and CIA mice were mounted on slides. The slides were blocked with 3% Bovine Serum Albumin for 1h and incubated with anti-ACACB (1:200, Cat# DF7980, Affinity), anti-PDK1 (1:200, Cat# DF4365, Affinity) or anti-XBP1 (1:200, Cat# sc-8015, Santa Cruz) primary antibodies at 4 °C for 12h, followed by staining with fluorescence-conjugated second antibodies. Iso-type-matched primary antibodies served as controls. The nuclei were stained with DAPI, and the coverslips were mounted using Antifade Mounting Medium (Beyotime). The images were acquired and analyzed using a Zeiss LSM 710 Confocal Imaging System (Zeiss, Oberkochen, Germany). Total fluorescence intensity was quantified using the ZEN 3.11 software. Graphs were generated by comparing the fluorescence intensities of CIA to normal mice samples. 6 biological replicates were performed.

### Statistical analysis

All data preprocessing, statistical analyses and plotting were completed with R 4.3.2 software. The Wilcoxon rank sum test was utilized for comparisons between two groups. The Pearson correlation analysis was used for correlations between two continuous variables, while the Spearman correlation analysis was used for correlations between ordered variables or non-normal distribution data. The data were represented as mean ± SEM. The student's t-test was performed between two groups, and the one-way ANOVA followed by Tukey's post hoc test was performed among multiple groups. The p-value of < 0.05 was considered statistically significant.

## Results

### Screening of Hub genes associated with fatty acid metabolic characteristics of RA synovium

The integrated gene expression matrix for the test dataset was obtained after applying the limma package for group data normalization and the sva package for batch effect removal. Before integrating the four datasets, the normalizeBetweenArrays function of the limma package was applied for intra-group data normalization (Fig. S2). Box plots and principal component analysis (PCA) graphs visualized the overall gene expression levels of the integrated datasets. Although the gene expression levels within the same dataset were generally consistent, there were still obvious batch effects between datasets from different platforms and different experiments (Fig. S2A and B). The batch effects were then removed using the ComBat function of the sva package (Fig. S2C and D), meeting the conditions for subsequent analysis. PCA analysis was performed on the sample grouping of disease objects RA and HC in the integrated dataset (Fig. S2E). The results showed that there were significant differences in gene expression between the RA and HC groups, implicating successful integration.

**Figure 1:**
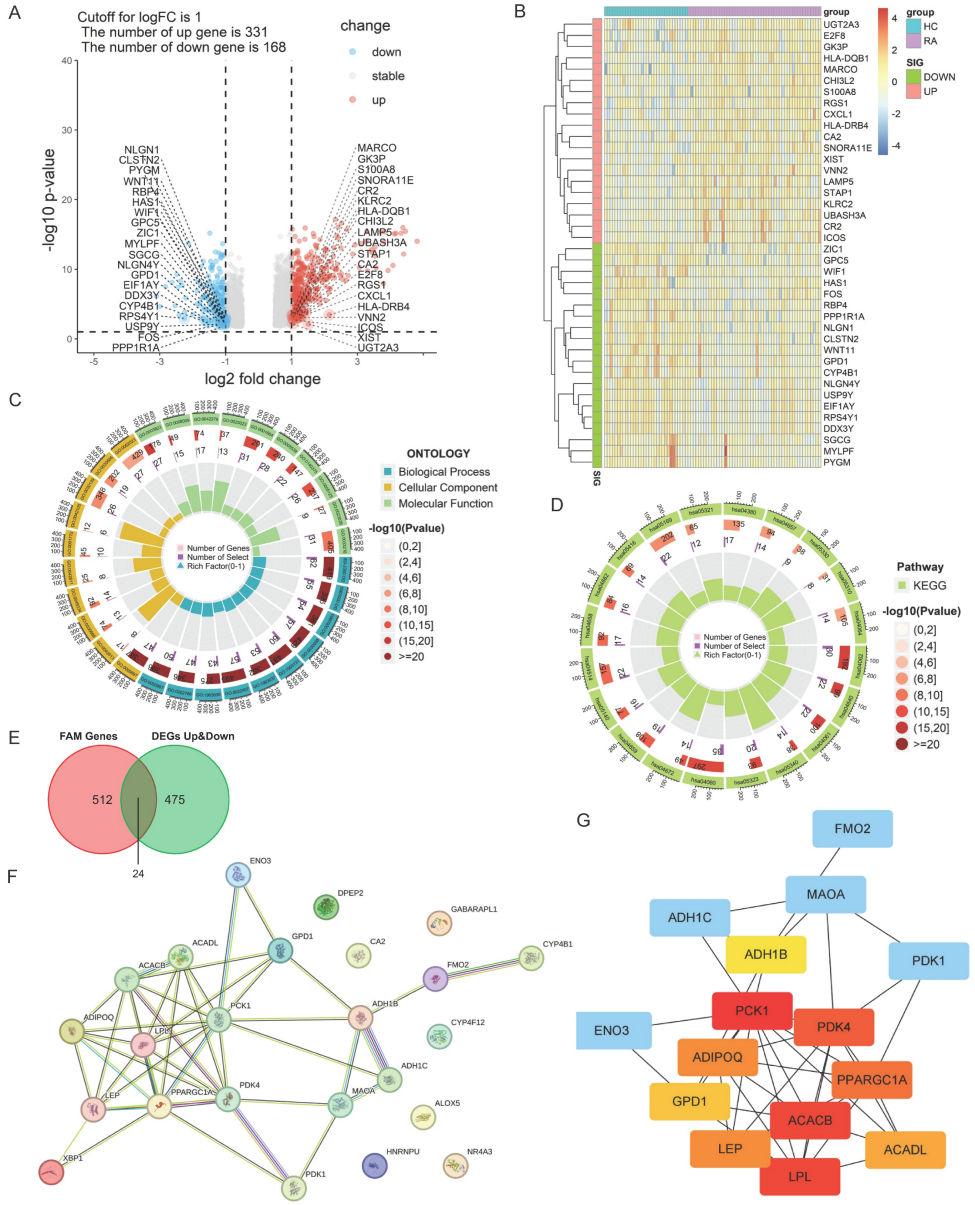
** Identification of differentially expressed genes (DEGs) between RA and HC. (A, B)** Volcano map and heatmap of DEGs. The top twenty significantly up-regulated DEGs (logFC > 1 and adj.p-value < 0.05) were labeled in red, and the top twenty significantly down-regulated DEGs (logFC < -1 and adj.p-value < 0.05) were labeled in blue. The color degree of the heatmap grid represents the gene expression. **(C, D)** GO and KEGG enrichment analysis of DEGs. GO ONTOLOGY displayed the top ten enrichment items. The top twenty enriched KEGG pathways were displayed. **(E)** Venn diagram of intersection of fatty acid metabolism-related (FAM-) genes and DEGs. **(F)** PPI network of DEGs related to fatty acid metabolic pathway. **(G)** Calculation of Hub candidate gene network based on CytoHubba method in Cytoscape. Node size represented connection number (degree) and node color represented mediated number centrality (blue: low mediated number centrality, red: high mediated number centrality).

The limma package was applied to conduct differential analysis on the integrated gene expression matrix. With the criteria of adj.p-value < 0.05 and |logFC| > 1, a total of 499 significantly differentially expressed genes (DEGs) were screened between synovium from patients with RA and HCs, including 331 up-regulated genes and 168 down-regulated genes (Fig. 1A and B). The GO and KEGG enrichment analyses for the DEGs showed that the biological processes for RA synovium DEGs were mainly enriched in the pathways related to leucocyte adhesion, monocyte differentiation, lymphocyte differentiation, cell-cell adhesion regulation, leukocyte migration, and T cell activation (Fig. 1C and D). The KEGG pathways were primarily concentrated in signaling interactions and the immune system, including cytokine and cytokine receptor interactions, chemokine signaling, hematopoietic lineage, cell adhesion molecules, and primary immunodeficiency disease pathways. Among these, the pathways associated with RA were mainly focused on Th17, Th1 and Th2 differentiation, osteoclast differentiation, IL-17 signaling, NF-κB signaling, and B cell receptor signaling pathways.

By intersecting the 499 DEGs with the 536 fatty acid metabolism-related (FAM-) genes selected from the MSigDB database, a total of 24 FAM-DEGs were identified as Hub candidate genes in RA synovium (Fig. 1E). The PPI network of FAM-DEGs was analyzed using the STRING database (Fig. 1F), and the Cytohubba plug-in of Cytoscape software was utilized to obtain the top ten Hub genes (Fig. 1G), among which *ACACB*, *PCK1*, *PDK4*, *PPARGGC1A* and *LPL* were central to the PPI network regulation.

### Immune cell infiltration characteristics and correlation analysis

**Figure 2:**
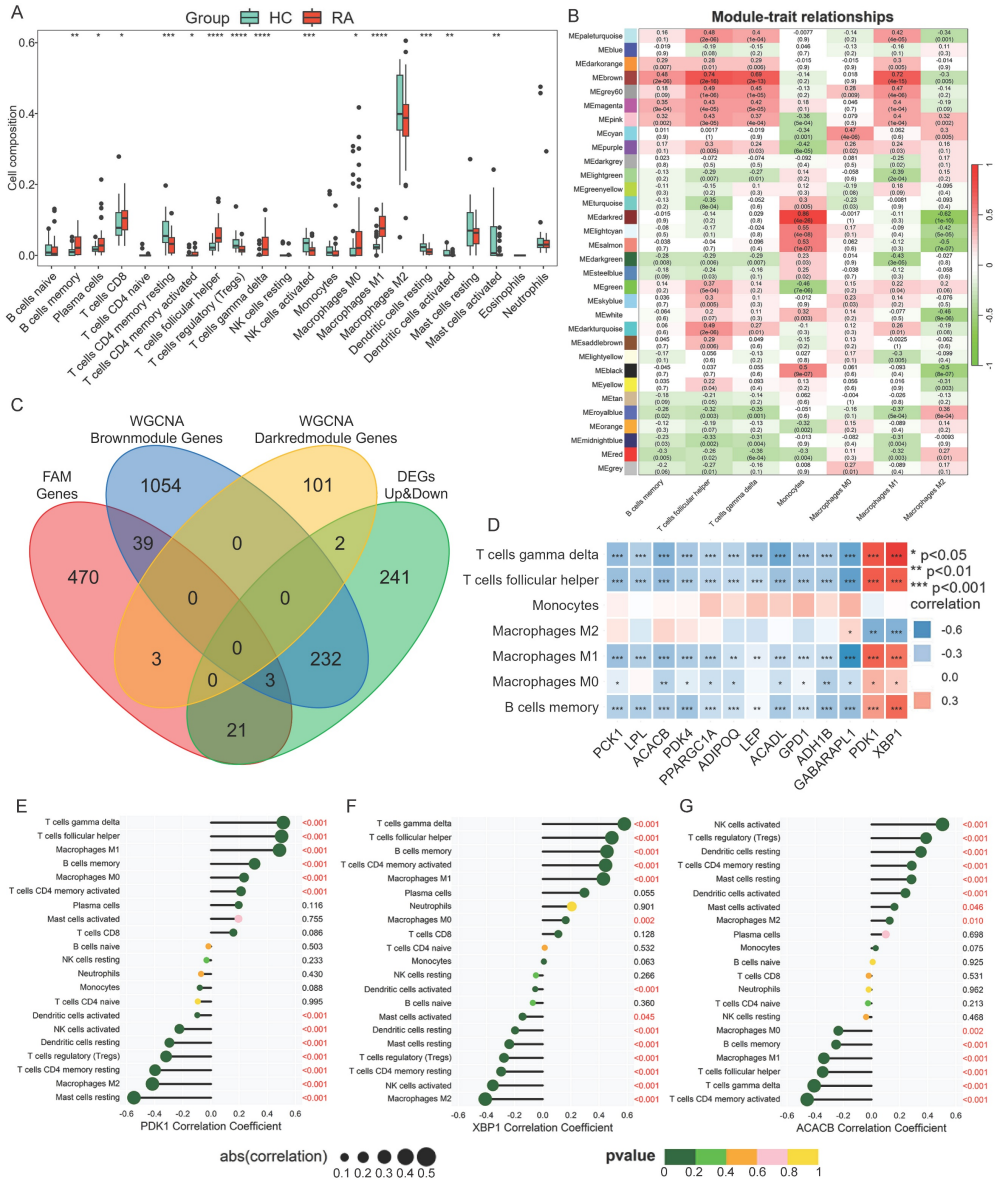
** WGCNA analysis of immune infiltrating cell phenotypes and their correlation with the Hub candidate genes. (A)** Box plot of significantly different immune cells between RA and HC. Analysis of synovial infiltrated immune cells based on CIBERSORT algorithm. The iterations number was 1000. The Wilcoxon test was utilized for statistics. **(B)** WGCNA of the infiltrating immune cell phenotypes. Relationship coefficient heatmap between gene modules and immune cells. **(C)**Venn diagram of intersection between module genes and DEGs associated with fatty acid metabolism. **(D)** Correlation between Hub candidate genes and immune cells.** (E-G)** Correlation between significant module genes and infiltrated immune cells via the Spearman test.

Immune cell infiltration analysis is critical for elucidating the mechanism of disease immune response and revealing the immune environment involved in RA synovium. The CIBERSORT algorithm was utilized to analyze the relative content of immune cells in the test dataset. The results of the immune cell infiltration analysis showed that the infiltration abundance of immune cells, including M0 and M1 macrophage, memory B cells, plasma cells, CD8 T cells, activated CD4 T cells, follicular helper T cells, and γδT cells, was higher in the synovial membrane of patients with RA compared to that in HCs (Fig. 2A). This indicated the existence of an overactivated immune microenvironment in RA synovium. Notably, not all these differences reached a threshold of statistical significance. This likely reflected the considerable biological heterogeneity of RA synovium, including variations in disease stage, treatment exposure, and the predominance of distinct synovial pathotypes. This pronounced inter-individual heterogeneity, combined with the limited sample size typical of bulk RNA-seq studies of human synovium, inherently reduced statistical power. The observed trend, however, still implicated a genuine biological shift, highlighting the complex and variable nature of RA synovitis.

The immune cells with significantly up-regulated infiltration capability in RA from the previous CIBERSORT analysis were used as the phenotypic data for WGCNA. Our study focused on the characteristics of the monocyte/macrophage lineage. Therefore, the phenotypes of immune cells included memory B cells, follicular helper T cells, gamma delta T cells, monocytes, and macrophages (M0, M1 and M2). In WGCNA, the Scale_Free_Topology_and_Mean, which showed scale-free fit index (y-axis) against different soft threshold (x-axis), was calculated (Fig. S3A-C).

When the threshold was defined as 0.9, the minimum soft threshold (sft$powerEstimate) was 9, which was selected as the optimal soft threshold for subsequent analysis. According to the moduled-trait relationship plot analyzed by WGCNA, the genes in the MEbrown and MEdarkred modules had the highest correlation with the phenotypes of immune cells, with the correlation coefficients of 0.74 and 0.86, respectively (Fig. 2B), indicating that there were genes highly associated with immune cell infiltration characteristics in RA synovium in these two modules. Therefore, the MEbrown and MEdarkred module genes were extracted and intersected with the FAM-genes and the DEGs. Three FAM-DEGs were extracted from the MEbrown module: *PDK1*, *XBP1* and *GABARAPL1* (Fig. 2C), and collected into the Hub candidate gene list. The correlation analysis between the total thirteen Hub genes and seven types of significantly different immune cells showed that the expression of *PDK1* and *XBP1* was positively correlated with γδT cells (*PDK1*, r = 0.51; *XBP1*, r = 0.58), follicular helper T cells (*PDK1*, r = 0.5; *XBP1*, r = 0.49), and M1 macrophages (*PDK1*, r = 0.49; *XBP1*, r = 0.43) (Fig. 2D). The Hub candidate gene *ACACB*, as screened by PPI network analysis, showed a negative correlation with M1 macrophages, γδT cells, and follicular helper T cells (Fig. 2D). Moreover, the significant correlation between* PDK1*, *XBP1* and *ACACB* and immune cell infiltration was confirmed using the Spearman test (Fig. 2E-G).

### Screening Hub genes based on machine learning algorithm

**Figure 3:**
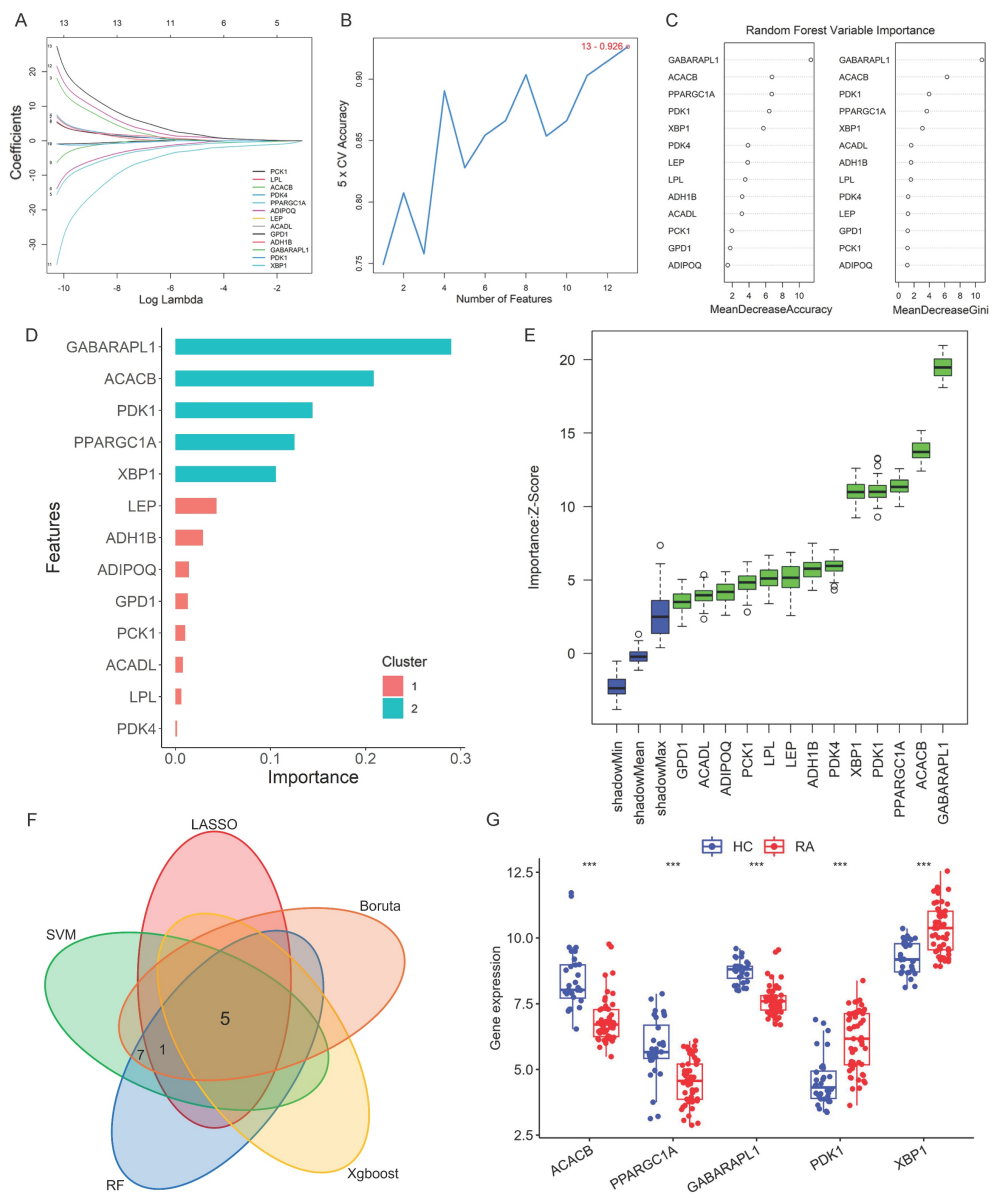
** Hub genes screening based on machine learning algorithms and validation. (A)** LASSO regression graph. **(B)** SVM-RFE feature-5-fold cross-validation accuracy diagram. **(C)** Importance ranking of Random Forest variables. **(D)** XGBoost analysis: Score results of importance of characteristic variables. **(E)** Boruta analysis. The box plot with green color represented the identified feature variables. **(F)** Intersection Venn diagram of five Hub genes screened by five machine learning algorithms. **(G)** Boxplot of the gene expression levels of *PDK1*, *XBP1* and *ACACB* for internal validation, and the Wilcoxon was utilized for statistics.

Five machine learning algorithms were employed to screen characteristic genes from the thirteen Hub candidate genes identified above. In the LASSO regression analysis, as the logarithm of the penalty coefficient (log Lambda) increased, the regression coefficients of all variables in the regression model gradually reduced (Fig. 3A). The model ultimately selected six feature variables, including *ACACB, PPARGC1A, ADIPOQ, GABARAPL1, PDK1* and* XBP1* (Supplementary Table S2). According to the SVM-RFE feature-5 cross-validation accuracy diagram, the accuracy of the thirteen genes analyzed was 0.915 (Fig. 3B). Random Forest (RF) analysis selected the optimal number of trees and determined the importance of the random forest variables (Fig. 3C). The XGBoost analysis of the importance scores of characteristic variables showed that the top five genes *GABARAPL1*, *ACACB*, *PDK1*, *PPARGC1A* and *XBP1* were crucial for distinguishing RA and HC diagnosis (Fig. 3D). The Boruta analysis screened the thirteen Hub candidate genes with higher scores than the shadow Max and identified them as feature genes (Fig. 3E). The Hub genes selected by the five machine learning algorithms were shown in Supplementary Table S2, respectively. The intersection of the screening results revealed five final Hub genes: *ACACB*, *GABARAPL1*, *PPARGC1A*, *PDK1*, and *XBP1* (Fig. 3F). The expression of these five genes was internally validated in the test dataset queue to evaluate the screening effect of the five machine learning algorithms. As shown in Figure 3G, the gene expression levels of *PDK1* and *XBP1* were significantly up-regulated, while the expression levels of* ACACB*, *GABARAPL1* and *PPARGC1A* were significantly down-regulated in samples from patients with RA.

### Diagnostic efficiency evaluation and temporal analysis for Hub genes

**Figure 4:**
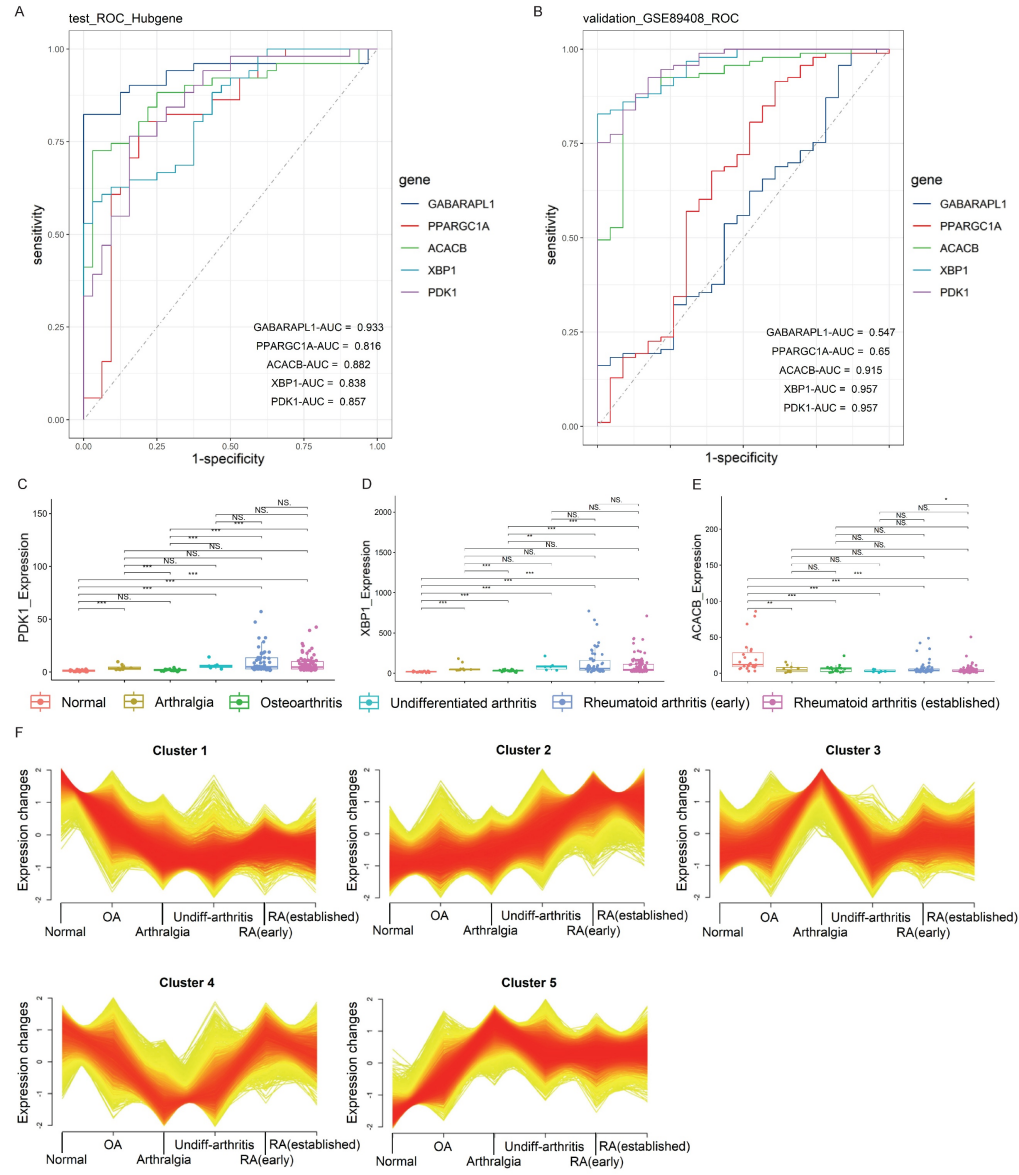
** Temporal analysis for the Hub candidate genes. (A, B)** Diagnostic ROC curves of internal and external validation of the Hub genes. The roc.test function was used for statistical difference analysis and diagnostic performance comparison. The area under the curves (AUCs) represented the diagnostic performance of the Hub genes. The closer the AUC value is to 1, the better the diagnostic performance.** (C-E)** *PDK1*, *XBP1* and *ACACB* expression levels in the validation dataset for external validation, and the Wilcoxon was utilized for statistics. **(F)** Clustering results of temporal analysis. *ACACB* belonged to Cluster 1; *PDK1* and *XBP1* belonged to Cluster 2. Also see Supplementary Table S3.

The diagnostic value of each gene from the five Hub genes (*ACACB*, *GABARAPL1*, *PPARGC1A*, *PDK1*, and *XBP1*) was evaluated by ROC analysis, with the RA diagnosis defined as the dependent variable and the expression level of each gene defined as the independent variable. The result showed that the AUC values of all five Hub genes were greater than 0.8 (Fig. 4A). Furthermore, external verification was conducted in the GSE89408 RAN-seq dataset, and the result showed that the AUC values of *PDK1*, *XBP1* and *ACACB* were all greater than 0.9 (Fig. 4B), indicating the abilities of these genes to accurately distinguish RA samples from HCs. However, the AUC values of *GABARAPL1* and *PPARGC1A* were less than 0.65, suggesting that their diagnostic values in different stages of RA were slightly inferior. Therefore, the Hub genes with the best diagnostic efficiency were *PDK1*, *XBP1* and *ACACB*.

The temporal analysis of gene expression patterns in GSE89408 RNA-seq validation dataset was conducted for the gene expression levels of *PDK1*, *XBP1* and *ACACB* across different disease stages, including normal, arthralgia, osteoarthritis, undifferentiated arthritis, RA (early), and RA (established). The results showed that the gene expression levels of *PDK1* and *XBP1* gradually increased, while the level of* ACACB* decreased in RA (Fig. 4C-E). Mfuzz analysis partitioned the dynamically expressed genes into 5 distinct temporal clusters (Fig. 4F). Among these, Cluster 1 and 2 displayed pronounced time-dependent patterns, characterized by a sustained downregulation and upregulation, respectively, over the course of the pseudo-development of RA. The clustering result indicated that the expression patterns of* ACACB* belonged to Cluster 1, and that of *PDK1* and *XBP1* belonged to Cluster 2 (Supplementary Table S3). The high membership values of these three Hub genes (Supplementary Table S4) strongly suggested their co-regulation within the specific temporal trajectories. These patterns were consistent with the alteration of FAM in response to RA progression. In summary, all three Hub genes displayed the characteristics of time-dependent changes corresponding to the disease progression, suggesting that these genes were important for the early onset and progression of RA.

### Targeted drug prediction for Hub genes

**Figure 5:**
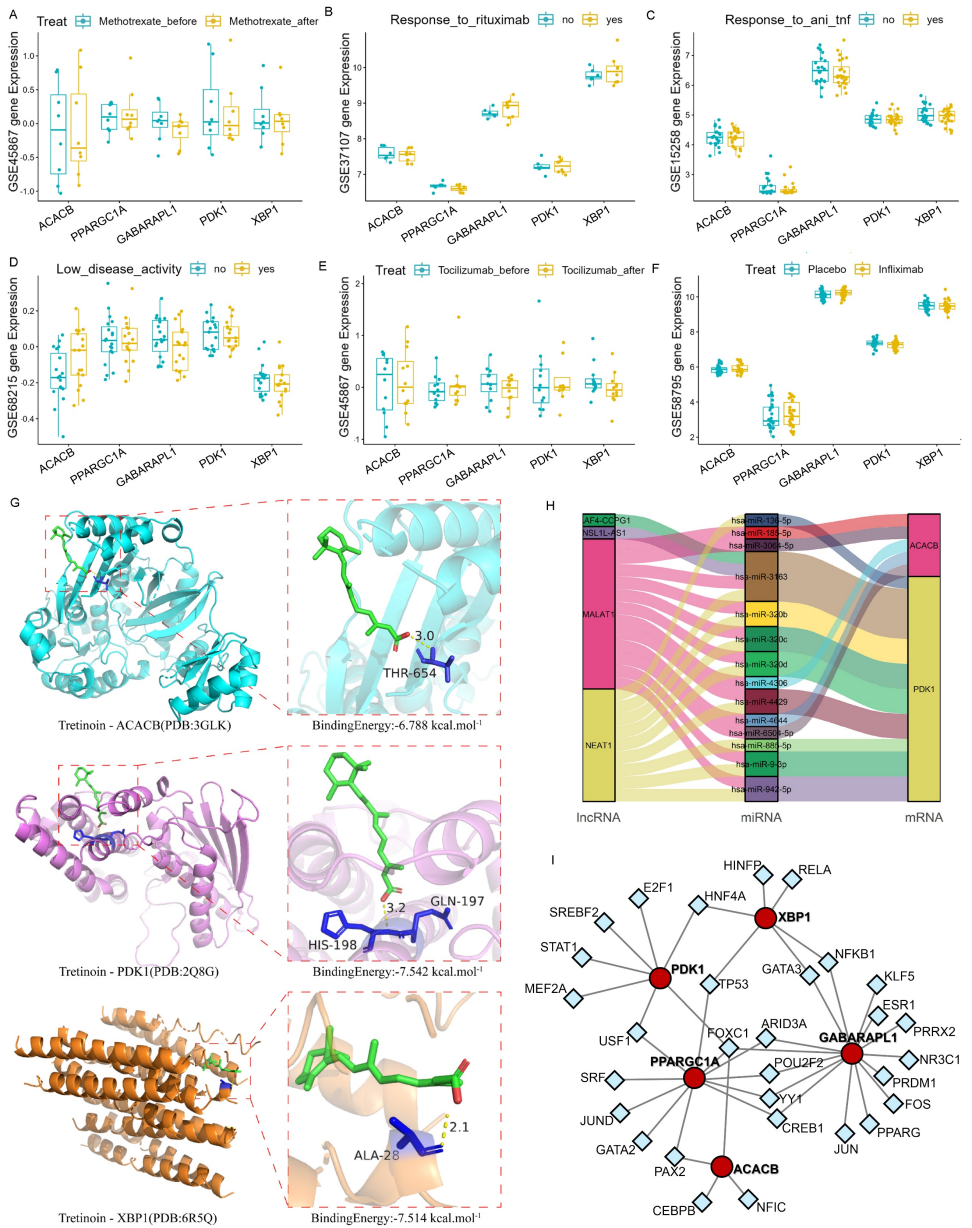
** Association between the Hub genes and RA. (A-F)** Expression levels of *PDK1*, *XBP1* and *ACACB* in the validation dataset with different immunotherapy treatments were used for immuno-efficacy evaluation. The Wilcoxon was utilized for statistics. **(G)** Molecular docking analysis of Tretinoin with the corresponding proteins of Hub genes. Three analysis results with the lowest binding energy were shown. **(H)** Mulberry map of lncRNA-miRNA-mRNA network interaction for *PDK1* and *ACACB*. **(I)** The interaction network between the Hub genes and the predicted transcription factor, with the red circle representing the Hub genes and the blue square representing the predicted transcription factors.

The immuno-efficacy of the Hub genes was evaluated using multiple datasets from mRNA isolated from the peripheral whole blood of patients with RA undergoing different immunotherapy treatments, including anti-TNF antibodies, Rituximab, Infiximab, Methotrexate, and Abatacept. The expression levels of the Hub genes showed no significant differences following the application of these drugs (Fig. 5A-F). Since joint inflammation and damage play a central role in RA development and the Hub genes were screened from synovial samples, analysis with tissue samples may yield positive results. However, there were few synovium datasets available for the immuno-efficacy evaluation.

The potential drug targets of these Hub genes were further explored using the DSigDB database, with the ranking results shown as binding force scores (Supplementary Table S5). Additionally, the AutoDock molecular docking was conducted to analyze the binding ability of Tretinoin to the corresponding proteins of the three Hub genes. The minimum binding energy of Tretinoin with PDK1, XBP1 and ACACB were -7.542 kcal/mol, -7.514 kcal/mol, and -6.788 kcal/mol, respectively (Fig. 5G), indicating highly stable binding of Tretinoin with all three proteins.

### ceRNA and transcription factor network prediction for Hub genes

miRNA induces gene silencing by down-regulating target mRNA expression through binding to mRNA, while upstream molecules circRNA and lncRNA regulate miRNA function by binding to miRNA response elements, thereby modulating mRNA expression. To further understand the regulatory network, interactions of lncRNAs and miRNAs with the mRNAs of the three Hub genes were predicted using miRDB (https://mirdb.org/index.html) and ENCORI (https://rnasysu.com/encori/) websites. No direct regulatory pathway was found for miRNA and upstream lncRNA targeting *XBP1* mRNA. However, MALAT1 and NEAT1 were identified as the main lncRNA-miRNA regulatory pathways for *PDK1* and *ACACB* (Fig. 5H). Meanwhile, the transcription factor prediction suggested that FOXC1 may be a common transcription factor regulating the expression of *PDK1* and *ACACB* (Fig. 5I).

### Hub gene expressions are regulated by proinflammatory cytokines

**Figure 6:**
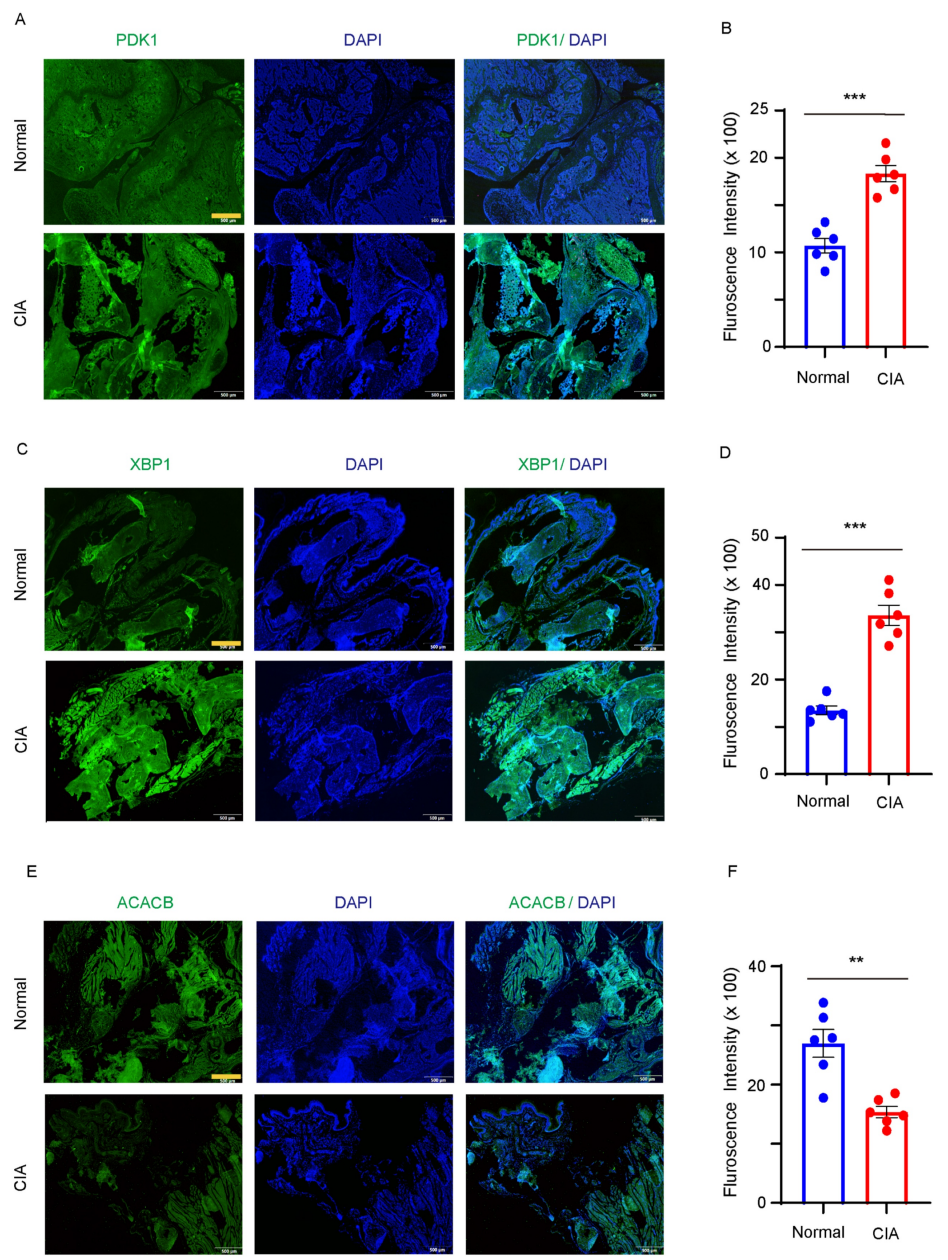
** PDK1, XBP1 and ACACB expression in synovial tissue**. DBJ mice were immunized with collage. **(A-B)** Immunofluorescence staining of PDK1 from the hind paw in vehicle and collage induced mice. Representative images were from one of six synovial tissue sections. Bar, 20µm. **(C-D)** Immunofluorescence staining of XBP1 from the hind paw in vehicle and collage induced mice. Representative images were from one of six synovial tissue sections. Bar, 20µm. **(E-F)** Immunofluorescence staining of ACACB from the hind paw in vehicle and collage induced mice. Representative images were from one of six synovial tissue sections. Bar, 20µm. All data are presented as the mean ± SEM. Paired Student t-test, ** *P* < 0.01; *** *P* < 0.001.

To confirm the abnormal expression of PDK1, XBP1 and ACACB in RA joint, a CIA mouse model for RA was utilized by injecting chicken type II collagen into DBJ mice. Immunofluorescent results showed significantly increased fluorescence intensities of PDK1(Fig. 6A and B) and XBP1(Fig. 6C and D), and decreased intensity of ACACB in the joint tissues of CIA mice compared to the vehicle treated mice (Fig. 6E and F).

**Figure 7:**
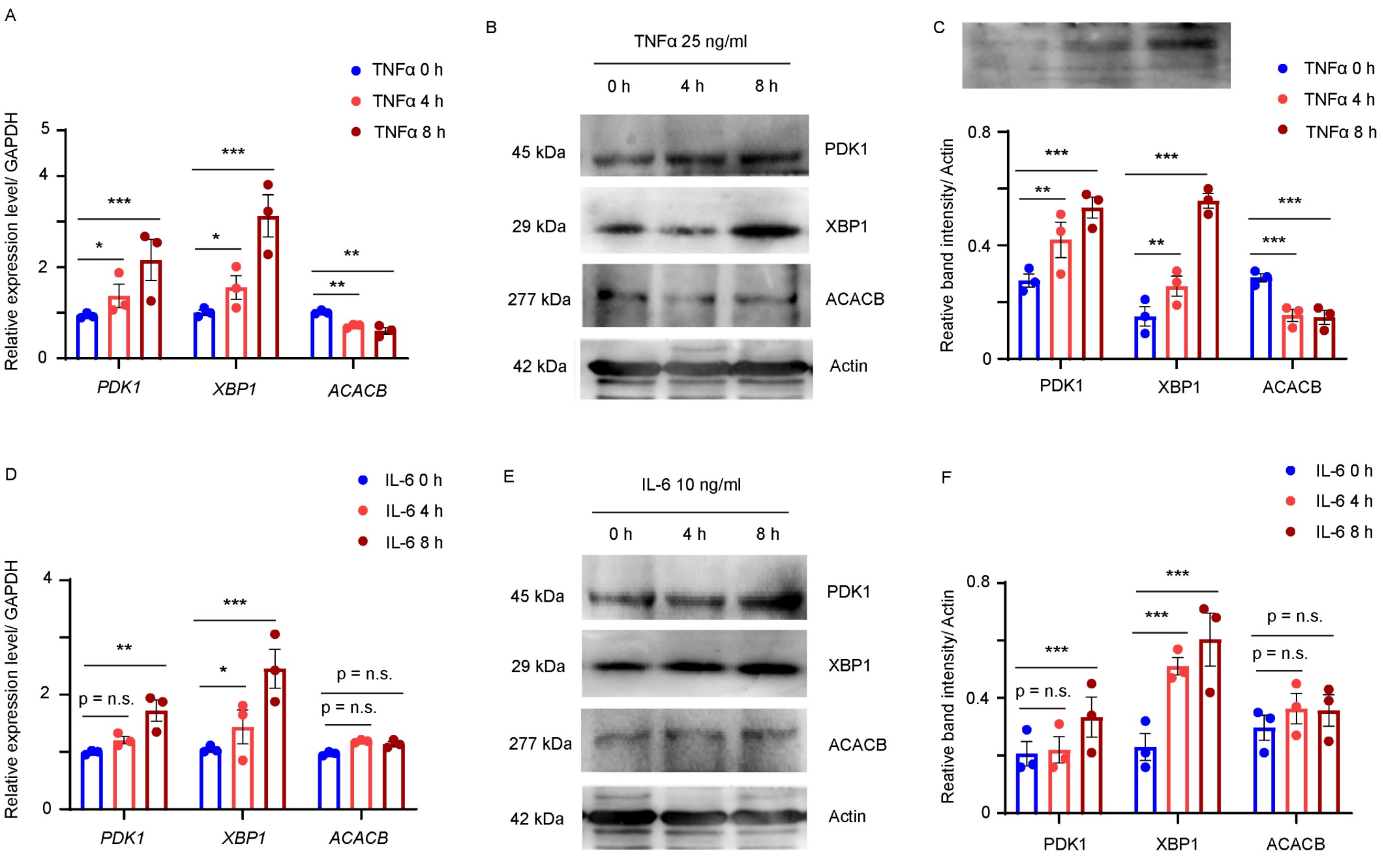
** PDK1, XBP1 and ACACB were regulated by proinflammatory cytokines in synovial fibroblasts. (A-C)** RA fibroblast-like synovial cells (FLS) MH7A were treated with vehicle or TNFα for indicated time. **(A)** Transcript levels of *PDK1*, *XBP1*, and *ACACB* were quantified by qPCR, biological replicates: n=3. **(B)** PDK1, XBP1 and ACACB expressions were detected by western blotting, representative images were shown. **(C)** Band intensity data from 3 replicate experiments were quantified. **(D-F)** MH7A cells were treated with vehicle or IL-6 for indicated time. **(D)** Transcript levels of *PDK1*, *XBP1*, and *ACACB* were quantified by qPCR, biological replicates: n=3. **(E)** PDK1, XBP1 and ACACB expressions were detected by western blotting, representative images were shown. **(F)** Band intensity data from 3 replicate experiments were quantified. All data are presented as the mean ± SEM. Paired Student t-test, * *P* < 0.05; ** *P* < 0.01; *** *P* < 0.001; n.s. no significance.

In RA, cells within the inflamed synovium produce various proinflammatory cytokines, particularly TNFα and IL-6. To assess whether increased TNFα and IL-6 levels in synovial tissue contributed to the abnormal expression of PDK1, XBP1 and ACACB in RA synovium, MH7A, a fibroblast-like synovial cell line generated from RA synovial tissue, was treated with 50 ng/mL TNFα or 10 ng/mL IL-6 for 4 or 8 hours, and the transcript levels of the three Hub genes were analyzed using qPCR. The result showed that the mRNA levels of *PDK1* and *XBP1* were significantly up-regulated and that of *ACACB* was down-regulated in the TNFα treated MH7A cells (Fig. 7A). Western blot confirmed that the bands intensities were increased for PDK1 and XBP1 and decreased for ACACB in the synovial fibroblast cells treated with TNFα (Fig. 7B and C). Similarly, higher expression levels of PDK1 and XBP1were induced by IL-6, while that of ACACB seemed to be unchanged after IL-6 treatment (Fig. 7D-F). Together, these findings indicated that the abnormal expression of PDK1, XBP1 and ACACB might be induced by inflammatory cytokines in RA synovium.

## Discussion

Fatty acid metabolism is highly associated with the pathogenesis of RA. In this study, using bioinformatics and machine learning algorithms, *PDK1*, *XBP1* and *ACACB,* which were related to fatty acid metabolic pathway, were identified as three Hub genes associated with immune cell infiltration into the RA synovial tissue and exhibited high diagnostic efficiency. These genes might be considered as significant risk factors in the onset and pathogenesis of RA.

In RA, fibroblast-like synoviocytes (FLSs) and immune cells interact in the synovium, leading to the activation of both cell types. Our analysis of immune cell infiltration showed that the most abundant cell types in the synovial membrane of RA were monocyte/macrophage, FLSs, and T lymphocytes. Moreover, memory B cells, plasma cells, and dendritic cells were also significantly increased in RA synovium. Consistently, multiple research suggested that several effector cells in the synovial membrane of RA could be potential therapeutic targets, including MERTK^+^ macrophages 44, NOTCH3^+^ synovial fibroblasts 45, CD11c^+^ autoimmune associated B cells 46, and PD-1^+^ peripheral helper T cells 47, 48. These findings indicate that these cells may play critical roles in the inflammatory environment formation of RA synovium. However, we should interpret these findings with caution. It is important to consider the methodological constraints of the CIBERSORT algorithm when applied to synovial tissue. First, CIBERSORT relies on a predefined signature matrix (LM22), which was built from circulating immune cells. Synovial tissue macrophages and fibroblasts can exist in unique activation states that may not be perfectly mirrored in the blood-derived signatures, potentially leading to misclassification or an inability to resolve specific tissue-resident subsets. Second, the computational deconvolution of bulk tissue RNA-seq data is inherently challenged by the similarity of gene expression profiles between different cell types. Activated synovial fibroblasts can express genes typical of myeloid cells, potentially leading to overestimation of certain immune populations. Finally, the hypoxic and inflamed synovial microenvironment can alter global gene expression profiles, which might not be fully accounted for in the reference matrix, introducing a potential source of bias.

Our study identified *PDK1*, *XBP1* and *ACACB* as the critical Hub genes related to fatty acid metabolic pathway, and these three Hub genes were significantly correlated with infiltrated M1 macrophages, γδT cells and follicular helper T cells in the synovium. A question arises is whether PDK1, XBP1 and ACACB are associated with synovium inflammatory environment. PDK1, a key kinase controlling fatty acid metabolism by phosphorylating pyruvate dehydrogenase subunits PDHA1 and PDHA2, regulates macrophage migration ability via HIF-1α-PDK1 axis 49. Moreover, lncRNA LOC100912373 promotes FLS proliferation by up-regulating PDK1 expression 50. XBP1, a major transcription factor regulating endoplasmic reticulum stress, can be activated by Toll like receptors in synovial fibroblasts from patients with active RA 51. Moreover, studies in ACC knockout mice have demonstrated that ACC/ACACB regulates acute inflammatory responses by limiting fatty *acid* oxidation in macrophage innate immunity 52. Our experiments using an RA mouse model confirmed the high expression of PDK1, XBP1 and lower expression of ACACB in the joints of CIA mice. These findings suggest that the three Hub genes may play important roles in the pathogenesis of RA synovium. Furthermore, temporal analysis demonstrated the time-dependent characteristics of *PDK1*, *XBP1* and *ACACB* in the development of RA. Considering that bone destruction in RA is an irreversible process and requires active intervention before entering the terminal stage, these genes may serve as potential disease indicators for early diagnosis of RA.

Immuno-efficacy results from molecular docking analysis successfully predicted the stable interaction between Tretinoin and the corresponding proteins of *PDK1*, *XBP1* and *ACACB*, indicating their potential as drug targets for RA treatment in terms of abnormal fatty acid metabolism. The analysis of peripheral blood datasets showed no significant differences in the expression of these genes in the efficacy evaluation of immunotherapy drugs. However, the three Hub genes identified here were from synovial datasets, yet there were few such datasets available for immuno-efficacy evaluation. Considering the difficulty for generating synovial datasets compared to peripheral blood datasets, future studies could first focus on revealing the molecular mechanisms and interactions between peripheral blood cells and synovial tissues, which might provide hints for hypothesis regarding immunotherapy efficacy on RA synovial membranes or fibroblasts. However, since immune responses, inflammation and tissue destruction primarily occurred in synovial tissues, further explorations for more comprehensive immune pathways and mechanisms with RA synovial samples and within the inflammatory microenvironment should be essential for more accurate immuno-efficacy analysis of the three Hub genes.

Meanwhile, the regulatory mechanism for PDK1, XBP1 and ACACB expression in synovial cells remains unclear. Although our studies showed that the MALAT1 and NEAT1 lncRNA-miRNA pathways and the transcription factor FOXC1 might play roles in the regulation of *PDK1* and *ACACB* expression, mechanistic studies are needed to illustrate the detailed regulatory pathways. In RA, cells within the synovium secrete various proinflammatory cytokines. Our results indicated that in the presence of TNFα or IL-6, the expression levels of PDK1, XBP1 seemed to be further increased, whereas ACACB expression was suppressed by TNFα. This suggests that the inflammatory microenvironment of RA synovium may be responsible for the exacerbation of abnormal fatty acid metabolism associated RA, implicating that the modified expression of PDK1, XBP1 and ACACB by inflammatory cytokines may further alter fatty acid metabolic pathways and promote RA development. Specifically, cytokines in the RA inflammatory microenvironment may upregulate PDK1 and XBP1 expression, and downregulate ACACB expression in the cells within synovium, altering the metabolic balance toward enhanced fatty acid oxidation. Such metabolic shift may meet the elevated energy requirement associated with inflammatory reactions and responses, promoting the inflammatory phenotypes of RA.

## Conclusion

In this study, we identified *PDK1*, *XBP1* and *ACACB* as the three Hub genes highly involved in fatty acid metabolism dysfunction and immune infiltration in RA synovium. These genes exhibited better diagnostic efficiency and showed characteristics of time-dependent changes in the course of RA pathogenesis. Further investigation successfully elucidated the abnormal expression of PDK1, XBP1 and ACACB in RA synovium, and suggested that their expression might be regulated by proinflammatory cytokines. These findings indicated the potential regulatory roles of these fatty acid metabolic genes in the inflammation of RA synovium.

## Supplementary Material

Supplementary figures and tables.

## Abbreviations

RA: Rheumatoid arthritis
FAM: Fatty acid metabolism
DEGs: Differentially Expressed Genes
PPI: Protein-Protein Interaction
GO: Gene Ontology
KEGG: Kyoto Encyclopedia of Genes Genomes
GSEA: Gene Set Enrichment Analysis
CIBERSORT: Cell-type Identification By Estimating Relative Subsets Of RNA Transcripts
WGCNA: Weighted Gene Co-expressed Network Analysis
ROC: Receiver Operating Characteristic curves
AUC: Area Under the Curve
LASSO: Least Absolute shrinkage and selection operator
SVM-RFE: Support Vector Machine Recursive Feature Elimination
RF: Random Forest
Xgboost: eXtreme Gradient Boosting
PDK1: Pyruvate Dehydrogenase Kinase Isoenzyme I
XBP1: X-box binding protein 1
ACACB: Acetyl-CoA carboxylase β subunit

## References

[1] Di Matteo A, Bathon JM, Emery P (2023). Rheumatoid arthritis. Lancet.

[2] McInnes IB, Schett G (2011). The pathogenesis of rheumatoid arthritis. N Engl J Med.

[3] Gibofsky A (2014). Epidemiology, pathophysiology, and diagnosis of rheumatoid arthritis: A Synopsis. Am J Manag Care.

[4] Luo TT, Wu YJ, Yin Q, Chen WG, Zuo J (2023). The Involvement of Glucose and Lipid Metabolism Alteration in Rheumatoid Arthritis and Its Clinical Implication. J Inflamm Res.

[5] Qiu J, Wu B, Goodman SB, Berry GJ, Goronzy JJ, Weyand CM (2021). Metabolic Control of Autoimmunity and Tissue Inflammation in Rheumatoid Arthritis. Front Immunol.

[6] Ryu H, Kim J, Kim D, Lee J-E, Chung Y (2019). Cellular and Molecular Links between Autoimmunity and Lipid Metabolism. Mol Cells.

[7] Aghakhani S, Soliman S, Niarakis A (2022). Metabolic reprogramming in Rheumatoid Arthritis Synovial Fibroblasts: A hybrid modeling approach. PLoS Comput Biol.

[8] Zuo J, Tang J, Lu M, Zhou Z, Li Y, Tian H (2021). Glycolysis Rate-Limiting Enzymes: Novel Potential Regulators of Rheumatoid Arthritis Pathogenesis. Front Immunol.

[9] Kraus FV, Keck S, Klika KD, Graf J, Carvalho RA, Lorenz H-M (2023). Reduction of Proinflammatory Effector Functions Through Remodeling of Fatty Acid Metabolism in CD8+ T Cells from Rheumatoid Arthritis Patients. Arthritis Rheumatol.

[10] Cai W-W, Yu Y, Zong S-Y, Wei F (2020). Metabolic reprogramming as a key regulator in the pathogenesis of rheumatoid arthritis. Inflamm Res.

[11] Mondal S, Saha S, Sur D (2024). Immuno-metabolic reprogramming of T cell: a new frontier for pharmacotherapy of Rheumatoid arthritis. Immunopharmacol Immunotoxicol.

[12] Takeuchi Y, Hirota K, Sakaguchi S (2019). Synovial Tissue Inflammation Mediated by Autoimmune T Cells. Front Immunol.

[13] de Jong TA, Semmelink JF, Denis SW, van de Sande MGH, Houtkooper RHL, van Baarsen LGM (2023). Altered lipid metabolism in synovial fibroblasts of individuals at risk of developing rheumatoid arthritis. Journal of Autoimmunity.

[14] Bustamante MF, Garcia-Carbonell R, Whisenant KD, Guma M (2017). Fibroblast-like synoviocyte metabolism in the pathogenesis of rheumatoid arthritis. Arthritis Research & Therapy.

[15] Yang Z, Matteson EL, Goronzy JJ, Weyand CM (2015). T-cell metabolism in autoimmune disease. Arthritis Research & Therapy.

[16] Zurier RB, Rossetti RG, Seiler CM, Laposata M (1999). Human peripheral blood T lymphocyte proliferation after activation of the T cell receptor: effects of unsaturated fatty acids. Prostaglandins Leukot Essent Fatty Acids.

[17] Anderson MJ, Fritsche KL (2004). Dietary polyunsaturated fatty acids modulate in vivo, antigen-driven CD4+ T-cell proliferation in mice. J Nutr.

[18] de Jong AJ, Kloppenburg M, Toes REM, Ioan-Facsinay A (2014). Fatty acids, lipid mediators, and T-cell function. Front Immunol.

[19] Lim SA, Wei J, Nguyen T-LM, Shi H, Su W, Palacios G (2021). Lipid signalling enforces functional specialization of Treg cells in tumours. Nature.

[20] Kanno T, Nakajima T, Kawashima Y, Yokoyama S, Asou HK, Sasamoto S (2021). Acsbg1-dependent mitochondrial fitness is a metabolic checkpoint for tissue Treg cell homeostasis. Cell Rep.

[21] Rodgers LC, Cole J, Rattigan KM, Barrett MP, Kurian N, McInnes IB (2020). The rheumatoid synovial environment alters fatty acid metabolism in human monocytes and enhances CCL20 secretion. Rheumatology (Oxford).

[22] Canavan M, Marzaioli V, McGarry T, Bhargava V, Nagpal S, Veale DJ (2020). Rheumatoid arthritis synovial microenvironment induces metabolic and functional adaptations in dendritic cells. Clin Exp Immunol.

[23] Wei J, Huang X, Zhang X, Chen G, Zhang C, Zhou X (2023). Elevated fatty acid beta-oxidation by leptin contributes to the proinflammatory characteristics of fibroblast-like synoviocytes from RA patients via LKB1-AMPK pathway. Cell Death Dis.

[24] Mateen S, Zafar A, Moin S, Khan AQ, Zubair S (2016). Understanding the role of cytokines in the pathogenesis of rheumatoid arthritis. Clin Chim Acta.

[25] Han C-K, Lee W-F, Hsu C-J, Huang Y-L, Lin C-Y, Tsai C-H (2021). DPP4 reduces proinflammatory cytokine production in human rheumatoid arthritis synovial fibroblasts. J Cell Physiol.

[26] Ma X, Xu S (2013). TNF inhibitor therapy for rheumatoid arthritis. Biomed Rep.

[27] Furst DE, Emery P (2014). Rheumatoid arthritis pathophysiology: update on emerging cytokine and cytokine-associated cell targets. Rheumatology (Oxford, England).

[28] Yokota K (2024). Osteoclast differentiation in rheumatoid arthritis. Immunol Med.

[29] Broeren MG, de Vries M, Bennink MB, Arntz OJ, Blom AB, Koenders MI (2016). Disease-Regulated Gene Therapy with Anti-Inflammatory Interleukin-10 Under the Control of the CXCL10 Promoter for the Treatment of Rheumatoid Arthritis. Hum Gene Ther.

[30] Woetzel D, Huber R, Kupfer P, Pohlers D, Pfaff M, Driesch D (2014). Identification of rheumatoid arthritis and osteoarthritis patients by transcriptome-based rule set generation. Arthritis Res Ther.

[31] Barrett T, Wilhite SE, Ledoux P, Evangelista C, Kim IF, Tomashevsky M (2013). NCBI GEO: archive for functional genomics data sets-update. Nucleic Acids Res.

[32] Huber R, Hummert C, Gausmann U, Pohlers D, Koczan D, Guthke R (2008). Identification of intra-group, inter-individual, and gene-specific variances in mRNA expression profiles in the rheumatoid arthritis synovial membrane. Arthritis Res Ther.

[33] Davis S, Meltzer PS (2007). GEOquery: a bridge between the Gene Expression Omnibus (GEO) and BioConductor. Bioinformatics.

[34] Ritchie ME, Phipson B, Wu D, Hu Y, Law CW, Shi W (2015). limma powers differential expression analyses for RNA-sequencing and microarray studies. Nucleic Acids Res.

[35] Guo Y, Walsh AM, Fearon U, Smith MD, Wechalekar MD, Yin X (2017). CD40L-Dependent Pathway Is Active at Various Stages of Rheumatoid Arthritis Disease Progression. J Immunol.

[36] Chen B, Khodadoust MS, Liu CL, Newman AM, Alizadeh AA (2018). Profiling Tumor Infiltrating Immune Cells with CIBERSORT. Methods Mol Biol.

[37] Newman AM, Liu CL, Green MR, Gentles AJ, Feng W, Xu Y (2015). Robust enumeration of cell subsets from tissue expression profiles. Nat Methods.

[38] Langfelder P, Horvath S (2008). WGCNA: an R package for weighted correlation network analysis. BMC Bioinformatics.

[39] Tibshirani R (1996). Regression Shrinkage and Selection via the Lasso. Journal of the Royal Statistical Society.

[40] Guyon I, Weston J, Barnhill S, Vapnik V (2002). Gene selection for cancer classification using support vector machines. Mach Learn.

[41] Chen T, Guestrin C (2016). XGBoost: A Scalable Tree Boosting System. ACM.

[42] Alexey N, Alois K (2013). Gradient boosting machines, a tutorial. Frontiers in Neurorobotics.

[43] Kursa MB, Rudnicki WR (2010). Feature Selection with Boruta Package. Journal of Statistical Software.

[44] Martínez-Ramos S, Rafael-Vidal C, Malvar-Fernández B, Pérez N, Mouriño C, Pérez SG (2023). Semaphorin3B promotes an anti-inflammatory and pro-resolving phenotype in macrophages from rheumatoid arthritis patients in a MerTK-dependent manner. Front Immunol.

[45] Chen J, Cheng W, Li J, Wang Y, Chen J, Shen X (2021). Notch-1 and Notch-3 Mediate Hypoxia-Induced Activation of Synovial Fibroblasts in Rheumatoid Arthritis. Arthritis Rheumatol.

[46] Bao W, Xie M, Ye Y (2022). Age-associated B cells indicate disease activity in rheumatoid arthritis. Cell Immunol.

[47] Cao G, Chi S, Wang X, Sun J, Zhang Y (2019). CD4+CXCR5+PD-1+ T Follicular Helper Cells Play a Pivotal Role in the Development of Rheumatoid Arthritis. Med Sci Monit.

[48] Yoshitomi H (2020). CXCL13-producing PD-1hiCXCR5- helper T cells in chronic inflammation. Immunol Med.

[49] Semba H, Takeda N, Isagawa T, Sugiura Y, Honda K, Wake M (2016). HIF-1alpha-PDK1 axis-induced active glycolysis plays an essential role in macrophage migratory capacity. Nat Commun.

[50] Fan C, Cui X, Chen S, Huang S, Jiang H (2020). LncRNA LOC100912373 modulates PDK1 expression by sponging miR-17-5p to promote the proliferation of fibroblast-like synoviocytes in rheumatoid arthritis. Am J Transl Res.

[51] Savic S, Ouboussad L, Dickie LJ, Geiler J, Wong C, Doody GM (2014). TLR dependent XBP-1 activation induces an autocrine loop in rheumatoid arthritis synoviocytes. J Autoimmun.

[52] Yeudall S, Upchurch CM, Seegren PV, Pavelec CM, Greulich J, Lemke MC (2022). Macrophage acetyl-CoA carboxylase regulates acute inflammation through control of glucose and lipid metabolism. Sci Adv.

