# Supplementary material for: Identification and Immune Landscape Analysis of Fatty Acid Metabolism-related Genes Associated with Rheumatoid Arthritis
Source: Int J Med Sci. 2026 Jan 1;23(2):389–405. doi: 10.7150/ijms.121972 (PMC12825009; doi:10.7150/ijms.121972)
Supplement: Supplementary file 1 — Supplementary figures and tables. [file ijmsv23p0389s1.pdf]

SUPPLEMENTARY INFORMATION

Supplemental MATERIAL AND METHOD

- Parameters and implementation details for Machine Learning algorithms

Figure.S1. The overall workflow of the bioinformatics analysis section

Figure.S2. Test dataset preprocessing and batch effect correction

Figure.S3. WGCNA and immune cell correlation analysis

Figure S4. Parameters and implementation details for machine learning algorithms

Table S1. The GEO datasets information included in this study

Table S2. The results of five machine learning algorithms screening

Table S3. The clustering results of temporal analysis

Table S4. The membership values of the Hub genes

Table S5. The top ten drugs from DSigDB prediction targeting the Hub genes

Supplemental MATERIAL AND METHOD

Parameters and implementation details for Machine Learning algorithms

All machine learning analyses were performed in the R environment (v4.3.2). The following packages and parameters were used to ensure reproducibility.

1. LASSO (Least Absolute Shrinkage and Selection Operator)

- **Package & Function:** glmnet package, cv.glmnet() and glmnet() functions.
- **Key Parameters:** The model was specified with family="binomial" for binary classification. The penalty type was set to L1 (LASSO) with alpha=1. A sequence of nlambda=1000 penalty (λ) values was evaluated.
- **Implementation & Results:** The regression coefficient path (Fig. 3A) illustrates how coefficients shrink towards zero as the penalty (λ) increases. The optimal λ was determined via 10-fold cross-validation (Fig. S4A). We selected the lambda.1se value (the largest λ within one standard error of the minimum binomial deviance) to obtain a robust and parsimonious model. This process identified 6 feature genes (Table S2) with non-zero coefficients.

2. SVM-RFE (Support Vector Machine - Recursive Feature Elimination)

- **Package & Function:** e1071 and caret packages, with a custom RFE routine.
- **Key Parameters:** A linear kernel was used. The RFE process was conducted with k=5 (for ranking) and halve.above=100. Model accuracy for feature subsets was evaluated via nfold=5 cross-validation.
- **Implementation & Results:** The relationship between the number of features and the 5-fold cross-validation accuracy is shown in Fig. 3B, while the corresponding error rate is in Fig. S4B. The subset of 13 features (Table S2) achieving the highest accuracy (0.926) and lowest error rate (0.0735) was selected for further analysis.

3. RF (Random Forest)

- **Package & Function:** randomForest package, randomForest() function.
- **Key Parameters:** An initial model with ntree=500 was built with importance= TRUE to calculate variable importance metrics. Analysis of the out-of-bag (OOB) error rate (Fig. S4C) indicated that the error stabilized with approximately 105 trees.
- **Implementation & Results:** A final model was built with the optimal ntree=105. Variable importance was measured by two metrics: MeanDecreaseAccuracy

and MeanDecreaseGini (Fig. 3C). 5 genes were considered significant and retained as feature genes (Table S2).

4. XGBoost (eXtreme Gradient Boosting)

- **Package & Function:** xgboost package, xgboost() function.
- **Key Parameters:** The model was trained for binary classification (objective ="binary:logistic"). Key hyperparameters included: a learning rate eta=0.3, maximum tree depth max_depth=6, row subsampling ratio subsample=0.7, column subsampling ratio colsample_bytree=0.7, L2 regularization lambda=1, and L1 regularization alpha =0.1. Training was performed for nrounds=1000 boosting rounds with early stopping after early_stopping_rounds=50 rounds without improvement.
- **Implementation & Results:** The model achieved a final training log-loss of 0.034905, indicating a strong fit. The analysis provided an importance-score (xgb_importance) for each feature, from which the top 5 genes were selected (Table S2).

5. Boruta

- **Package & Function:** Boruta package, Boruta() function.
- **Key Parameters:** The analysis was run with a confirmed significance level of pValue=0.01, using mcAdj=TRUE (Bonferroni correction), for a maximum of maxRuns=500 iterations. Progress was monitored with doTrace=2.
- **Implementation & Results:** The algorithm iteratively compared the importance of original features with shadow features. The line plot (Fig. S4D) shows the convergence of the algorithm, and the final output (Fig. 3E) confirms feature genes in green color. All 13 candidate hub genes (Table S2) were confirmed as significant, as their importance scores exceeded the maximum score of the shadow features (shadowMax).

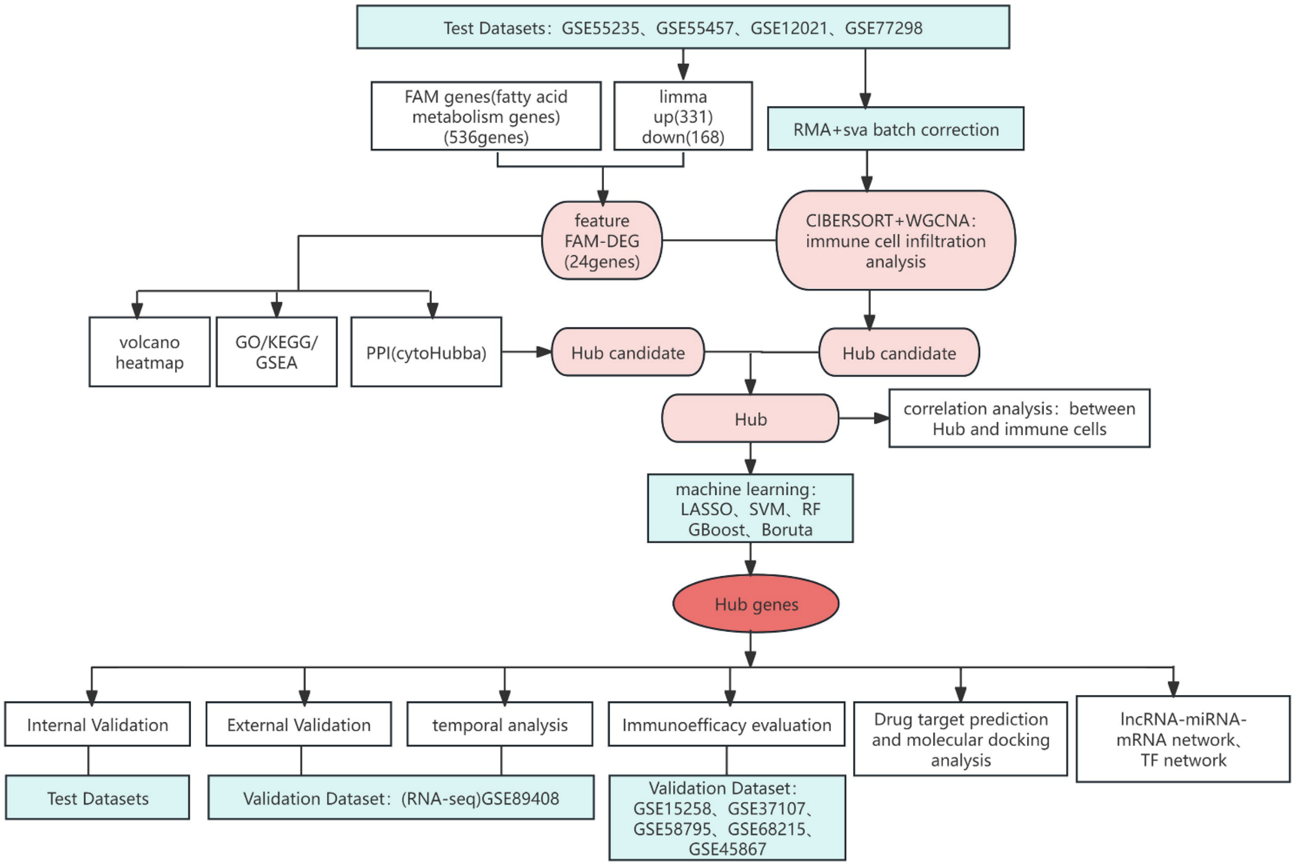

Figure S1. The overall workflow of the bioinformatics analysis section. This study screened Hub genes related to fatty acid metabolism in RA synovium by analyzing mRNA microarray data, investigating the expression patterns of Hub genes during RA disease and characterizing immune cells infiltration to synovium tissue by means of protein-protein interaction network analysis, immune infiltration analysis, temporal analysis, immuno-efficacy evaluation, drug prediction and ceRNA and transcription factor network analysis.

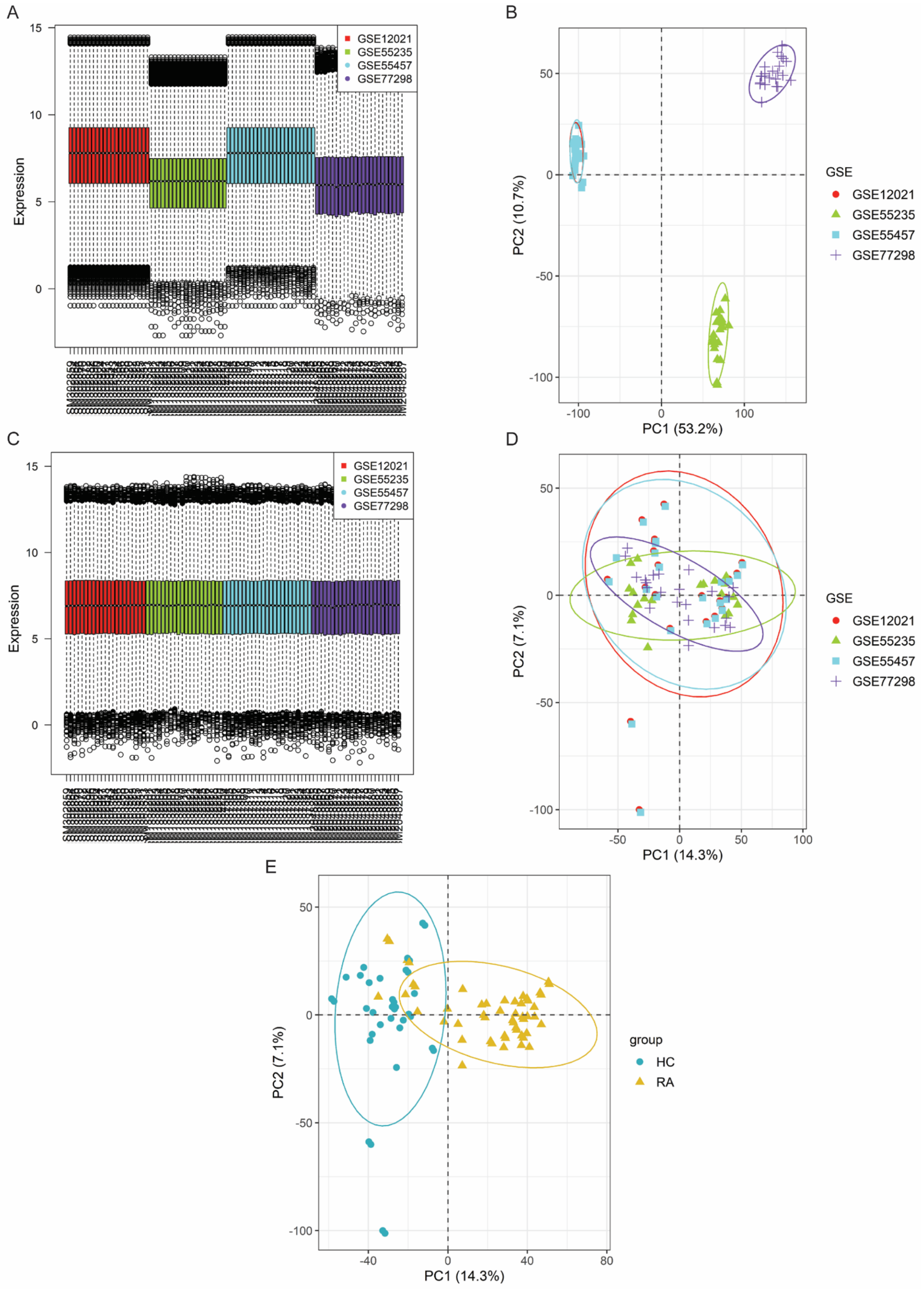
A
B
C
D
E
Expression
PC1 (53.2%)
PC2 (10.7%)
PC1 (14.3%)
PC2 (7.1%)
GSE
GSE12021
GSE55235
GSE55457
GSE77298
group
HC
RA

Figure S2. Test dataset preprocessing and batch effect correction. Four microarray datasets GSE12021, GSE55235, GSE55457 and GSE77298 expression matrices were integrated into the test dataset. **(A, B)** Boxplots before and after batch effect correction. **(C, D)** PCA plots before and after batch effect correction. The confidence ellipse showed the distribution of samples from different datasets with 95% confidence level. **(E)** PCA plot for RA and HC.

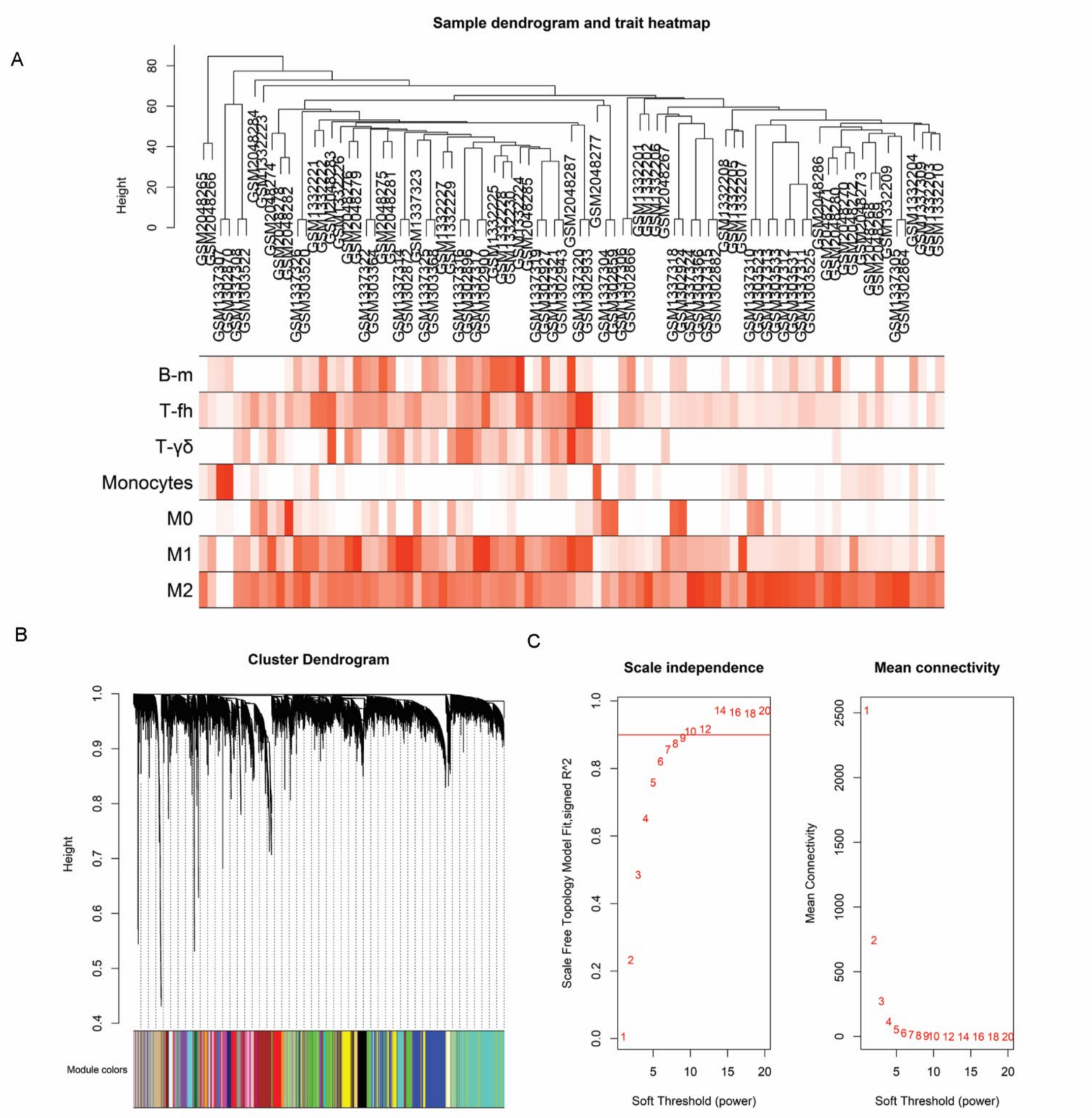

Figure S3. WGCNA and immune cell correlation analysis. (A-C) WGCNA analysis. **(A)** Sample clustering tree. The top half was sample clustering, the bottom half was phenotypic clustering, red represented samples from patients with RA, white represented samples from HCs. **(B)** Genes with similar expression patterns were clustered, different colors are different gene clusters. **(C)** Optimal soft threshold power.. The horizontal coordinate represented the weight parameter, and the vertical coordinate in the left figure was the square R2 of the

correlation coefficients of log(k) and log(p(k)) in the corresponding network. The higher the value, the closer the network was to the distribution without network scale, and the vertical coordinate in the right figure was the mean value of all gene adjacency functions in the corresponding gene module.

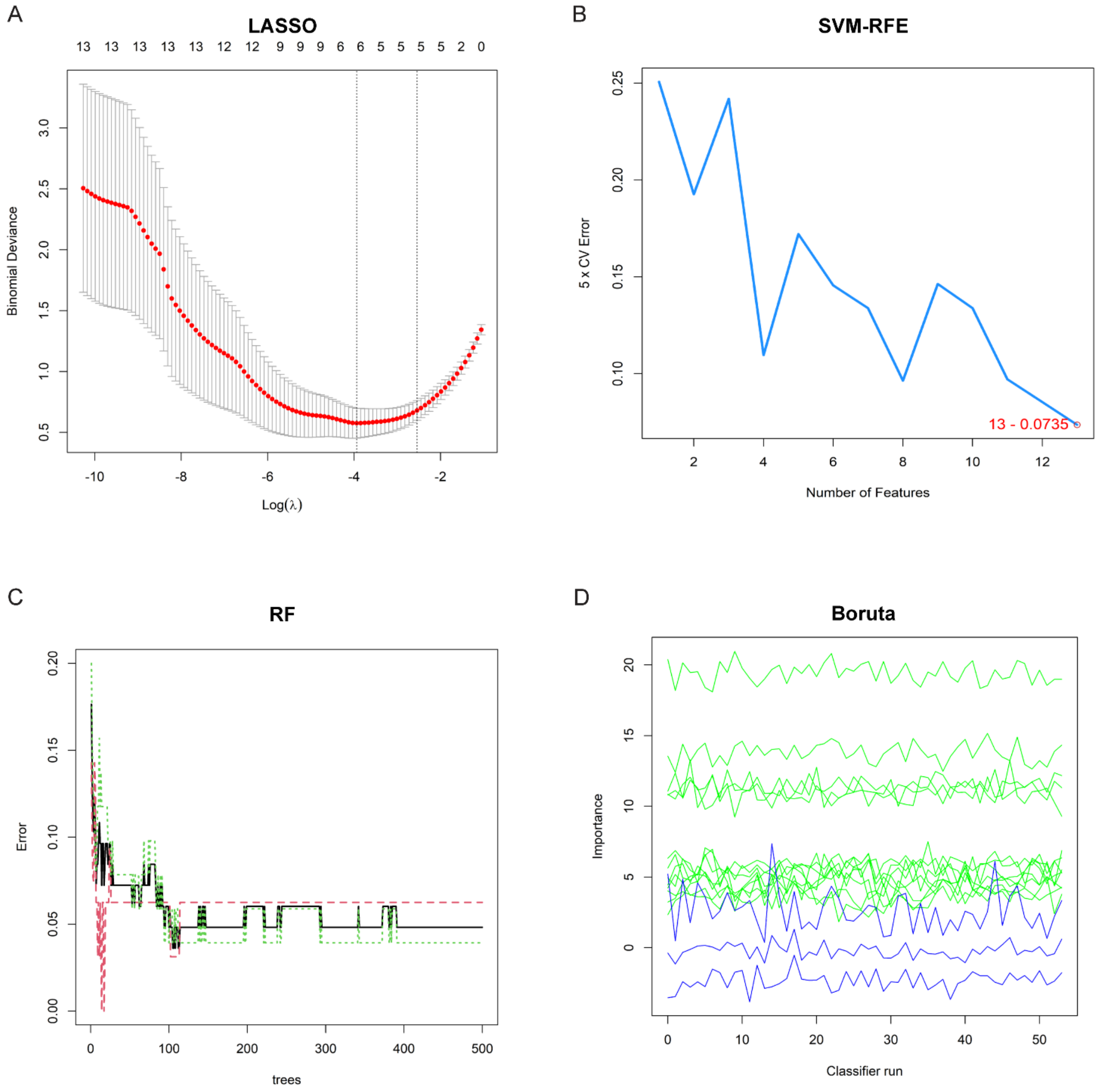

Figure S4. Parameters and implementation details for machine learning algorithms. (A) The LASSO regression curve graph. nfold = 10, family = "binomial", type.measure = "deviance". **(B)** SVM-RFE feature -5-fold cross-validation error rate relationship graph. nfold = 5. **(C)** The random forest plot of the RF analysis. ntree = 500; optimal ntree = 105. **(D)** The Boruta analysis line graph. pValue = 0.01, mcAdj = TRUE, maxRuns = 500, doTrace = 2.

Table S1. The GEO datasets information included in this study.

Attribute	GEO/ Platform	Tissue	Samples	Experiment type	Reference
test	GSE12021 GPL96	Synovial	21(9HC+12RA)	Array	Huber R
test	GSE55235 GPL96	Synovial	20(10HC+10RA)	Array	Woetzel D
test	GSE55457 GPL96	Synovial	23(10HC+13RA)	Array	Woetzel D
test	GSE77298 GPL570	Synovial	23(7HC+16RA)	Array	Broeren MG
validation	GSE89408 GPL11154	Synovial	173(23HC+57earlyRA+93establishedRA+18OA+10Arthralgia+6Undifferentiated arthritis)	RNAseq	Walsh AM, Guo Y
validation	GSE15258 GPL570	whole blood	46anti-TNF treatment (22noresponse+24response)	Array	Bienkowska JR
validation	GSE37107 GPL6947	whole blood	14 anti-rituximab treatment (6noresponse+8response)	Array	Raterman HG
validation	GSE58795 GPL10379	whole blood	59 anti-TNF treatment (29placebo+30infliximab)	Array	MacIsaac KD
validation	GSE68215 GPL4133	whole blood	36methotrexate/abatacept treatment (17Low disease activity+19no Low disease activity)	Array	Derambure C
validation	GSE45867 GPL57	whole blood	12Tocilizumab treatment (12before+12after) 16Methotrexate treatment (8before+8after)	Array	Ducreux J

Table S2. The results of five machine learning algorithms screening.

Rank	LASSO	SVM-RFE	RF	Xgboost	Boruta
1	*ACACB*	*GABARAPL1*	*GABARAPL1*	*GABARAPL1*	*PCK1*
2	*PPARGC1A*	*XBP1*	*ACACB*	*ACACB*	*LPL*
3	*ADIPOQ*	*ADIPOQ*	*PPARGC1A*	*PDK1*	*ACACB*
4	*GABARAPL1*	*ACADL*	*PDK1*	*PPARGC1A*	*PDK4*
5	*PDK1*	*PCK1*	*XBP1*	*XBP1*	*PPARGC1A*
6	*XBP1*	*PPARGC1A*			*ADIPOQ*
7		*PDK1*			*LEP*
8		*ACACB*			*ACADL*
9		*GPD1*			*GPD1*
10		*PDK4*			*ADH1B*
11		*ADH1B*			*GABARAPL1*
12		*LPL*			*PDK1*
13		*LEP*			*XBP1*

Table S3. The clustering results of temporal analysis.

Gene	Normal	OA	Arthralgia	UnA	RA (early)	RA (established)	cluster
ACACB	22.776	6.065	5.741	2.964	7.055	4.435	1
PDK1	1.260	1.816	4.200	6.494	9.980	7.526	2
XBP1	17.815	31.165	68.014	91.410	132.818	93.916	2
GABARAPL1	14.370	17.653	23.989	19.390	14.537	16.954	3
PPARGC1A	0.834	0.718	1.280	1.361	0.666	0.932	5

FPKM values are presented. Abbreviations: **OA**, Osteoarthritis; **UnA**, Undifferentiated arthritis.

Table S4. The membership values of the Hub genes.

Cluster	1	2	3	4	5
ACACB	**0.869377210**	0.01233196	0.01503514	0.09476897	0.008486721
PDK1	0.008302751	**0.89648370**	0.02152320	0.01805064	0.055639703
XBP1	0.013553311	**0.79217883**	0.04214648	0.02769070	0.124430675

Membership value threshold: > 0.5.

Table S5. The top ten drugs from DSigDB prediction targeting the Hub genes.

**Drug names**	**P-value**	**Adjusted P-value**	**Combined Score**	**Genes**
Tretinoin CTD 00006918	0.009644542	0.040943812	219193.0129	*ACACB, PDK1, XBP1*
Rimonabant hydrochloride CTD 00003133	0.002398159	0.038930571	4018.416231	*XBP1*
bupropion CTD 00007131	0.002547917	0.038930571	3729.253306	*XBP1*
Nilotinib CTD 00004428	0.00269766	0.038930571	3476.150033	*XBP1*
5-Nitroso-8-quinolinol CTD 00004584	0.002847389	0.038930571	3252.888057	*XBP1*
SU-6668 MRC	0.003446153	0.038930571	2574.276639	*PDK1*
IN1541 CTD 00001481	0.003446153	0.038930571	2574.276639	*ACACB*
L-sorbose CTD 00006006	0.003595807	0.038930571	2443.769793	*ACACB*
Go 7874 MRC	0.003595807	0.038930571	2443.769793	*PDK1*
lasalocid PC3 UP	0.003745445	0.038930571	2324.863325	*XBP1*
